# Modeling *Drosophila* gut microbe interactions reveals metabolic interconnectivity

**DOI:** 10.1016/j.isci.2021.103216

**Published:** 2021-10-06

**Authors:** Jürgen W. Schönborn, Fiona A. Stewart, Kerstin Maas Enriquez, Irfan Akhtar, Andrea Droste, Silvio Waschina, Mathias Beller

**Affiliations:** 1 Institut für Mathematische Modellierung Biologischer Systeme, Heinrich-Heine-Universität Düsseldorf, 40225 Düsseldorf, Germany; 2 Systembiologie des Fettstoffwechsels, Heinrich-Heine-Universität Düsseldorf, 40225 Düsseldorf, Germany; 3 Christian-Albrechts-University Kiel, Institute of Human Nutrition and Food Science, Nutriinformatics, Heinrich-Hecht-Platz 10, 24118 Kiel, Germany

**Keywords:** Microbial metabolism, Microbiome

## Abstract

We know a lot about varying gut microbiome compositions. Yet, how the bacteria affect each other remains elusive. In mammals, this is largely based on the sheer complexity of the microbiome with at least hundreds of different species. Thus, model organisms such as *Drosophila melanogaster* are commonly used to investigate mechanistic questions as the microbiome consists of only about 10 leading bacterial species. Here, we isolated gut bacteria from laboratory-reared *Drosophila*, sequenced their respective genomes, and used this information to reconstruct genome-scale metabolic models. With these, we simulated growth in mono- and co-culture conditions and different media including a synthetic diet designed to grow *Drosophila melanogaster*. Our simulations reveal a synergistic growth of some but not all gut microbiome members, which stems on the exchange of distinct metabolites including tricarboxylic acid cycle intermediates. Culturing experiments confirmed our predictions. Our study thus demonstrates the possibility to predict microbiome-derived growth-promoting cross-feeding.

## Highlights

• Reconstruction of six *Drosophila* gut bacteria genome-scale models
• Gut bacteria consortium modeling reveals growth interdependencies
• Certain *Drosophila* gut bacteria show growth-promoting cross-feeding
• Experimental validation of the modeling-derived growth-promoting effect

## Graphical abstract

**Figure:**
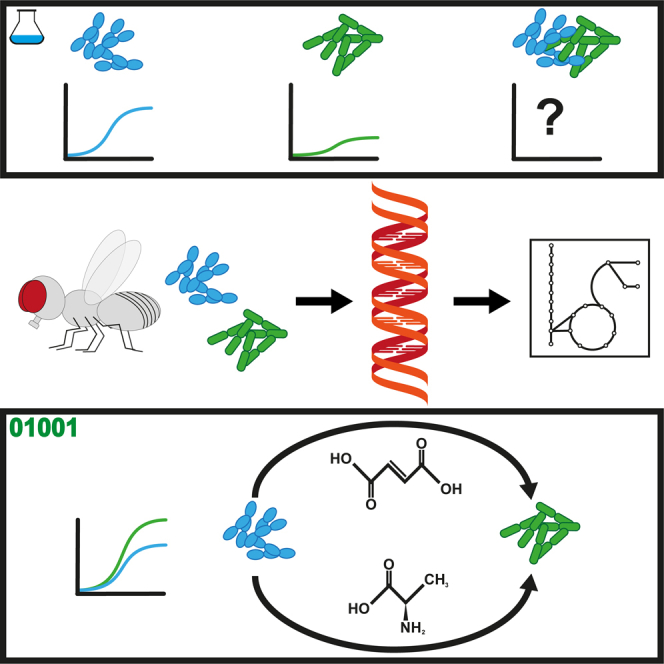

## Introduction

Multicellular organisms are inhabited by a vast number of microorganisms, which is generally termed the microbiome. In humans, the number of associated bacteria is in the same range as the cells of the host (Sender et al., 2016). As an entity, the bacteria encode an overwhelming number of genes and thus expand the metabolic capabilities of the host enormously. We are still at the beginning of understanding this metabolic interplay. Yet, first reports demonstrated an importance of the microbes present in the gut, the so-called gut microbiome, in humans and model organisms for increasing nutrient availability and energy harvest (Krajmalnik-Brown et al., 2012), the production of important bioactive metabolites including branched-chain amino acids (Lin et al., 2017; Liu et al., 2020), the metabolism of pharmaceuticals applied to the host (Clayton et al., 2009; Haiser et al., 2013), or the release of metabolites which affect signaling pathways of the host (Martin et al., 2019; Shin et al., 2011). Thus, the microbiome affects the host far beyond nutrient access. The importance of the gut microbiome can be seen most prominently in times of a perturbation or altered microbiome composition, which has been linked to many human diseases such as diabetes (Hartstra et al., 2014; Komaroff, 2016), obesity (Tilg and Kaser, 2011; Turnbaugh and Gordon, 2009), autism (Vuong and Hsiao, 2016), or inflammatory bowel disease (Halfvarson et al., 2017). Based on the observation that a perturbed microbiome is linked to pathologies, microbiome-focused therapies appear possible. Indeed, microbiome transfer therapies proved effective for the treatment of infections with the pathogen *Clostridium difficile* (Weingarden et al., 2013) and many pro- and prebiotic dietary regimens are already used (Arora et al., 2013).

The microbiome of mammals with hundreds to thousands of different bacterial species is extremely complex. In addition, many of these species cannot be cultured *ex vivo*, which hinders detailed functional analyses. Simpler model organisms can help to overcome these limitations and thus provide access to targeted functional analyses. The microbiome of *Drosophila melanogaster*, for example, only consists of 5–20 different species (Douglas, 2019; Ludington and Ja, 2020), which makes it much easier to analyze. Still, the gut microbiome of *Drosophila* has a significant impact on many aspects of the hosts' life such as the survival under nutrient limiting conditions, the lifespan of the flies, or the locomotor behavior (Consuegra et al., 2020a; Keebaugh et al., 2018; Ridley et al., 2012; Schretter et al., 2018; Shin et al., 2011; Silva et al., 2020; Storelli et al., 2011, 2018). The most abundant *Drosophila* gut bacteria belong to the *Lactobacilli, Acetobacter*, and *Enterococci* genera. Key species of these bacteria are culturable under standard laboratory conditions (Adair et al., 2018; Broderick and Lemaitre, 2012; Erkosar et al., 2013).

The prominent *Drosophila* gut microbiome members *Lactobacillus plantarum* and *Lactobacillus brevis* are Gram-positive rod-shaped lactic acid-producing microaerophilic bacteria from the Firmicutes phylum, which promote the systemic growth of fly larvae under nutrient-limiting conditions (Storelli et al., 2011). In humans, several lactobacilli strains have been shown to confer host health benefits (Marco et al., 2010), and a decline in their abundance is commonly associated with diseases (Aron-Wisnewsky et al., 2020; Heeney et al., 2018; Lee et al., 2020; Schwarzer et al., 2016). *Acetobacter* in contrast are Gram-negative, acetic acid-producing bacteria within the class of alpha-proteobacteria. They can be isolated from a variety of sources such as fruits and flowers and are often used to generate fermented food, e.g., vinegar (Azuma et al., 2009). *Acetobacter* species are major constituents of the *Drosophila* gut microbiome. Like lactobacilli (Storelli et al., 2011), they contribute to a successful larval development under nutrient-limiting conditions (Shin et al., 2011). This growth-promoting effect was demonstrated to stem on the secretion of acetic acid, which interferes with the insulin signaling pathway of the fly (Shin et al., 2011). This observation underpins the importance of secreted metabolites in terms of an interaction not only with the host but also likely with other members of the gut microbiome. At this point, the beneficial as well as detrimental (e.g., in terms of competition for nutrients) interactions between the microbiome members are not clear. First analyses, however, detected a complex interplay between combinations of the bacterial species and the host, which shapes host fitness through life history trade-offs (Gould et al., 2018). Similarly, also studies with isolated bacteria using growth on agar-based solid media (Sommer and Newell, 2018) or chemically defined media (Aumiller et al., 2021) support growth-promoting effects among the bacterial species of the *Drosophila* gut microbiome.

In order to investigate such metabolic interactions, we isolated bacteria from laboratory-reared *Drosophila* and investigated their isolated growth in different media such as *Lactobacillus*-promoting MRS and *Acetobacter*-selective ACE media. Furthermore, we used a synthetic diet suitable to grow *D. melanogaster* (holidic *Drosophila* diet; HD) (Piper et al., 2014). Six bacterial strains were analyzed in total and we resequenced their respective genomes to reconstruct genome-scale metabolic networks. These were used in single and co-culture growth simulations using the BacArena software package (Bauer et al., 2017). Our results reveal co-operative growth of certain bacteria based on the exchange of distinct metabolites including tricarboxylic acid cycle (TCA) intermediates, certain sugars, as well as amino acids in the D- and L-form. In analogous growth experiments, we could confirm the growth-promoting effect of several identified metabolites. Thus, the simulations open the door to systematically investigate the metabolic interplay of gut microbiome constituents and to reveal beneficial metabolites, which can promote the growth of selected gut microbiome constituents.

## Results

### Bacterial isolation, species identification, and *in vitro* growth characteristics

We started our analysis with the isolation of bacteria from the intestine of *white[-]* and Oregon-R adult flies (see material and methods). First, we isolated in total six morphologically distinct colonies on either *Lactobacillus* growth-promoting MRS- or *Acetobacter*-enriching ACE-agar plates and subsequently extracted the respective genomic DNA of our pure cultures. The 16S rRNA gene region of all clones was amplified by PCR, subcloned, and sequenced to allow species identification by BLAST searches. In total, we isolated two *L. plantarum*, one *L. brevis*, two *Acetobacter indonesiensis*, and one *Acetobacter pasteurianus* strains (see Table 1).

**Table 1:** Sequencing results, genome reassembly, and generated genome-scale model summaries

	*L. plantarum* (A2)	*L. plantarum* (B2)	*L. brevis* (B6)	*A. indonesiensis* (A4)	*A. indonesiensis* (A5)	*A. pasteurianus* (B5)
**Genome assembly**						

Reads [#]	3,587,296	3,638,786	3,277,616	3,319,800	3,387,326	3,239,340
Used reads [#]	2,902,970	3,125,105	1,917,270	2,060,518	2,036,898	2,077,587
Used reads [%]	86.2	90.3	59.6	62.2	60.3	66.9
Unmapped [#]	495,436	353,339	1,324,152	1,254,692	1,345,613	1,070,761
Genes [#]	3,676	3,559	2,595	3,352	3,364	3,091
Ref. genome sequence length (bp)	3,581,586	3,581,586	2,340,228	3,396,180	3,396,180	3,007,920
Reference genome	BDGP2	BDGP2	ATCC 367	NBRC 16471	NBRC 16471	BDGP5

**Metabolic models**						

Reactions [#]	1,815	1,815	1,584	1,931	1,931	1,796
Metabolites [#]	1,567	1,567	1,411	1,763	1,763	1,673
Genes [#]	657	658	473	631	632	580
Blocked reactions [%]	40.1	40.1	41	43.3	43.3	43.7
Unbalanced reactions [%]	9.4	9.4	10.2	8.2	8.2	8.5
Exchange reactions [%]	9	9	9.9	7.5	7.5	7.7

Bacterium	Isolate	Ref. genome	NCBI ID	Link
*Acetobacter pasteurianus*	B5	BDGP5	ASM245613v1	https://www.ncbi.nlm.nih.gov/assembly/GCF_002456135.1/
*Acetobacter indonesiensis*	A4	NBRC 16471	ASM799107v1	https://www.ncbi.nlm.nih.gov/assembly/GCF_007991075.1/
*Acetobacter indonesiensis*	A5	NBRC 16471	ASM799107v1	https://www.ncbi.nlm.nih.gov/assembly/GCF_007991075.1/
*Lactobacillus plantarum*	A2	BDGP2	ASM229018v1	https://www.ncbi.nlm.nih.gov/assembly/GCF_002290185.1/
*Lactobacillus plantarum*	B2	BDGP2	ASM229018v1	https://www.ncbi.nlm.nih.gov/assembly/GCF_002290185.1/
*Lactobacillus brevis*	B6	ATCC 367	ASM1446v1	https://www.ncbi.nlm.nih.gov/assembly/GCF_000014465.1/

The upper part of the table summarizes the sequencing results in terms of the number of reads obtained for the six bacterial resequencing reactions. These sequencing results were mapped with the ASA³P software (Schwengers et al., 2020) to the respective reference genomes whose ID as well as NCBI accession is provided. The details of the mapping results in terms of the number and percent of used (as well as unmapped) reads, the number of detected genes, and the genome sequence length are provided. The resequenced genome sequences were subsequently used to build the genome-scale metabolic models (see materials and methods). The lower part of Table 1 provides the details of the six genome-scale models in terms of the number of reactions, metabolites, mapped genes, blocked and unbalanced, as well as exchange reactions. All sequencing, ASA^3^P, and model data are available at https://doi.org/10.17632/2tgjd6y4zb.1.

**Figure 1:**
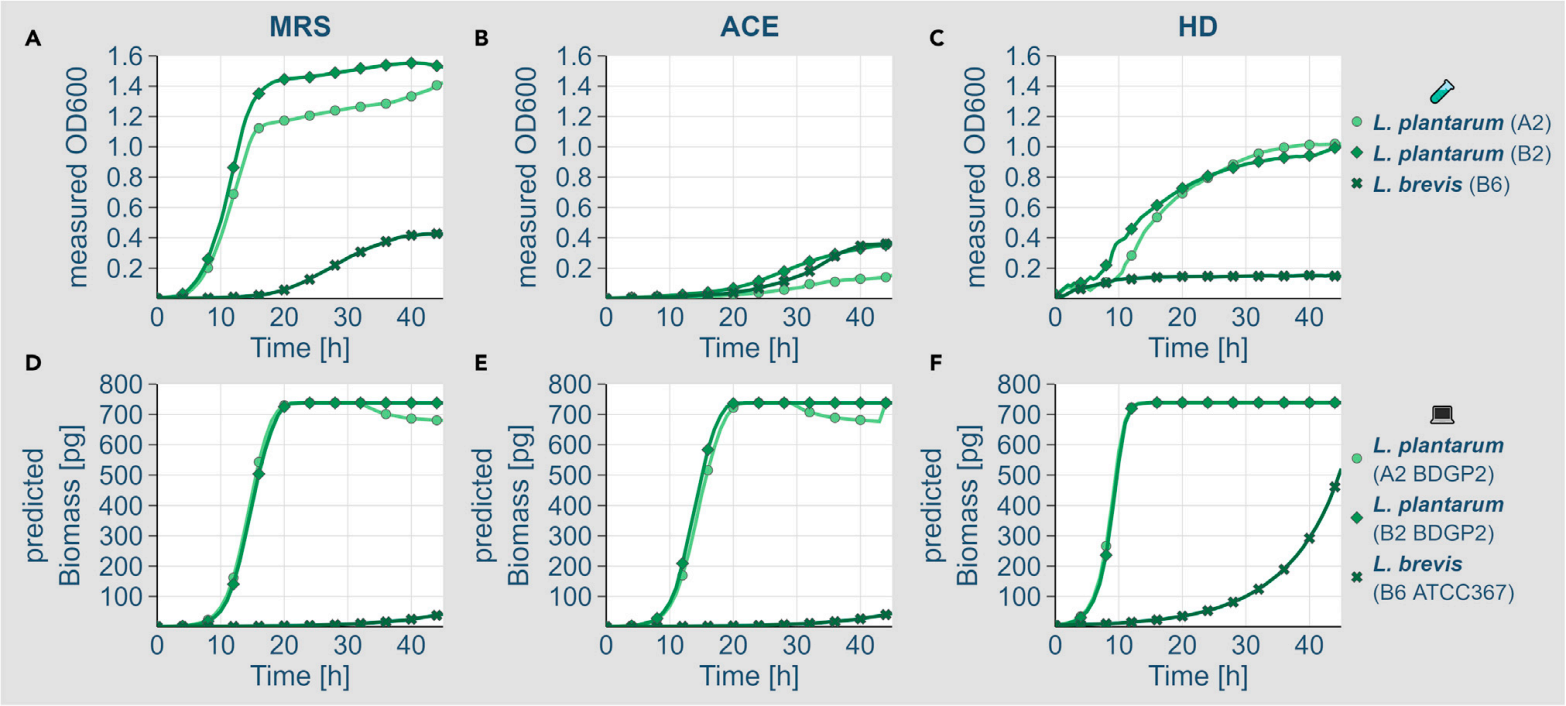
Wet-lab and *in silico* growth of *Lactobacillus* on different media (A–C) Growth of the *Lactobacillus* isolates *L. plantarum* (A2, light green, dot), *L. plantarum* (B2, medium green, check), and *L. brevis* (B6, dark green, cross) on MRS (A), ACE (B), and HD (C) media. Growth of all bacteria was monitored for at least 45 h in a plate reader without shaking. All cultures were inoculated with a 1:1,000 dilution for MRS and ACE media and with a 1:100 dilution for the HD medium. All cultures had an optically dense pre-culture. Representative growth curves of at least three biologically independent experiments are shown. Growth curves show mean values of triplicate measurements. (D–F) Simulated growth of the same bacteria in the same media as shown in (A–C). For the isolated bacteria, the genomes were resequenced and used to reconstruct genome-scale metabolic networks. These were used for growth simulations using the BacArena software package (Bauer et al., 2017) in combination with MRS (D), ACE (E), and HD (F) media. *L. plantarum* (A2, light green, dot), *L. plantarum* (B2, medium green, check), and *L. brevis* (B6, dark green, cross) on MRS (D), ACE (E), and HD (F) media. The simulations for each bacterium were run at least 12 times, and the computed growth curves represent the mean values. Detailed model data are available at https://doi.org/10.17632/2tgjd6y4zb.1. Wet-lab (A–C; small reaction tube) and *in silico* data (D–F; computer) are also indicated by the pictograms and labels on the right side of the figure.

**Figure 2:**
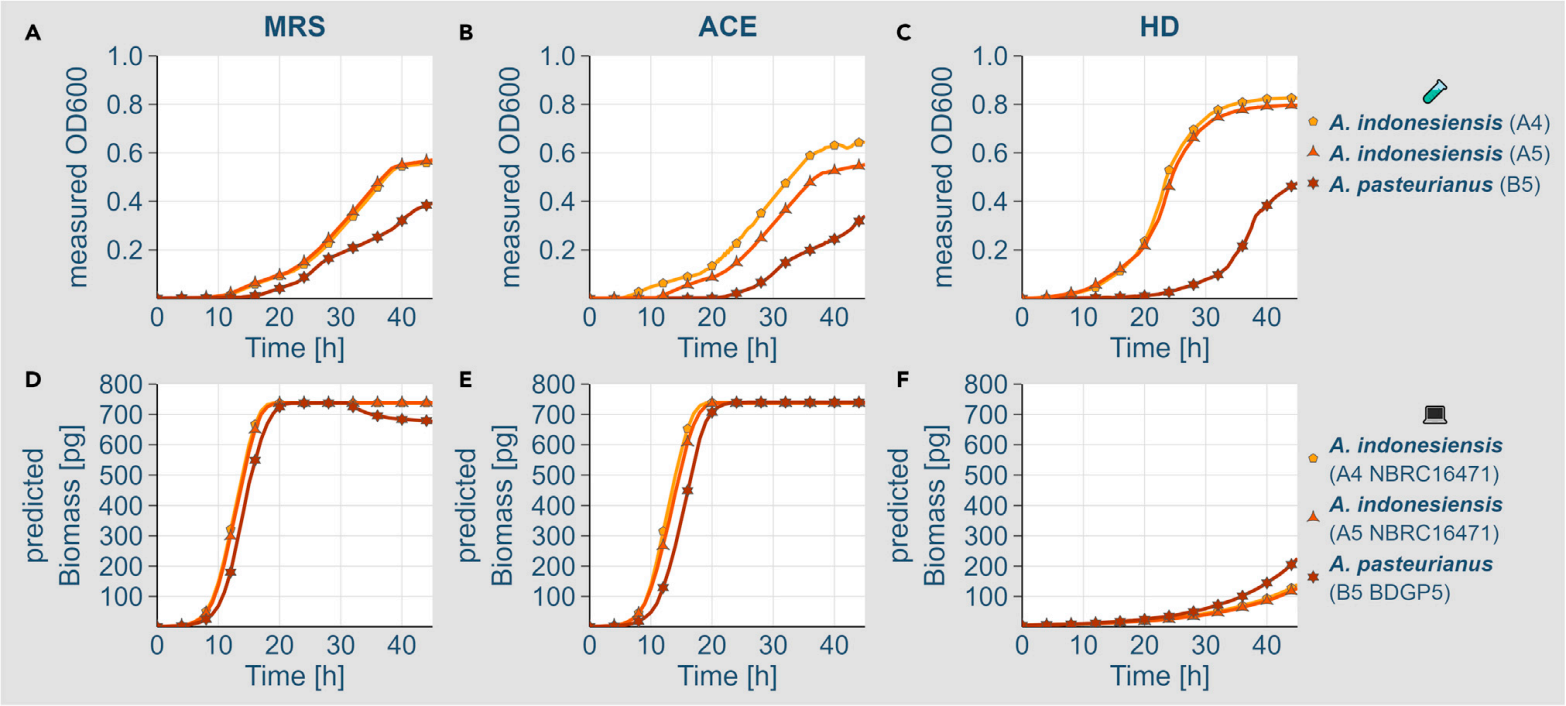
Wet-lab and *in silico* growth of *Acetobacter* on different media (A–C) Growth of the *Acetobacter* isolates *A. indonesiensis* (A4, light orange, pentagon), *A. indonesiensis* (A5, medium orange, triangle), and *A. pasteurianus* (B5, dark orange, star) on MRS (A), ACE (B), and HD (C) media. Growth of all bacteria was monitored for at least 45 h in a plate reader with shaking. All cultures were inoculated with a 1:1,000 dilution for MRS and ACE media and with a 1:100 dilution for the HD medium. All cultures had an optically dense pre-culture. Representative growth curves of at least three biologically independent experiments are shown. Growth curves show mean values of triplicate measurements. For the isolated bacteria, the genomes were resequenced and used to reconstruct genome-scale metabolic networks. These were used for growth simulations using the BacArena software package (Bauer et al., 2017) in combination with MRS (D), ACE (E), and HD (F) media. (D–F) *A. indonesiensis* (A4, light orange, pentagon), *A. indonesiensis* (A5, medium orange, triangle), and *A. pasteurianus* (B5, dark orange, star) on MRS (D), ACE (E), and HD (F) media. The simulations for each bacterium were run at least 12 times, and the computed growth curves represent the mean values. Detailed model data are available at https://doi.org/10.17632/2tgjd6y4zb.1. Wet-lab (A-C; small reaction tube) and *in silico* data (D-F; computer) are also indicated by the pictograms and labels on the right side of the figure.

We tested next the growth of the different bacteria in three different growth media (Figures 1 and 2). On top of the commonly used semi-defined MRS (*Lactobacillus* enriching medium; see materials and methods) and ACE (promoting *Acetobacter* growth; see materials and methods) liquid culturing media, we also tested for growth in a chemically defined (holidic diet [HD]) growth medium sufficient to culture *D. melanogaster* (Piper et al., 2014). All isolated lactobacilli were able to grow on the MRS medium (Figure 1A). *L. brevis*, however, showed a lower total growth than the two *L. plantarum* isolates (Figure 1A). On the ACE medium, all lactobacilli only showed low growth (Figure 1B) demonstrating the selectivity of the growth medium. In line with previous results (Consuegra et al., 2020a), *L. plantarum* grew relatively well on the HD, whereas *L. brevis* again only showed a low growth (Figure 1C). To our surprise, growth of the *Acetobacter* isolates did not differ much on the MRS and ACE media (Figures 2A and 2B). *A. indonesiensis* isolates showed prominent growth on the HD (Figure 2C). *A. pasteurianus*, in contrast only showed a relatively poor growth on the HD (Figure 2C). An overview of the experimentally determined growth rates is provided as Figure S1.

The determination of growth of single species cultures is trivial, whereas the determination of the individual contribution of distinct species to the biomass production of a consortium is difficult. Yet, a better understanding of the mutual effect on the growth of bacterial consortia is an intriguing and important question. Modeling experiments are a possibility to overcome this obstacle. For the modeling, an exact knowledge of the nutritional content of the growth medium is very important. Thus, growth of the bacteria on HD was particularly important, as this diet allows the exact description of the input for the modeling experiments. In the past, we already benefitted from this for modeling the growth and metabolism of *Drosophila* larvae (Schönborn et al., 2019). In order to reconstruct the genome-scale metabolic networks of the isolated bacteria, our next step was to sequence their respective genomes using the Illumina MiSeq platform (see material and methods). In the following, the genomes were assembled using whole-genome information as a scaffold, which we obtained from the NCBI database.

### Sequencing of the isolate genomes and model reconstruction

The sequencing runs resulted in 3.2–3.6 Mio reads per genome (see Table 1). The reads were mapped to the whole-genome sequences of *L. plantarum* BDGP2, *L. brevis* ATCC367, *A. indonesiensis* NBRC16471, and *A. pasteurianus* BDGP5, respectively, and further analyzed using the ASA³P software (Schwengers et al., 2020) (the complete dataset is available in the supplement). Between 60% and 90% of the total reads mapped to the respective reference strains (see Table 1).

We reconstructed the genome-scale metabolic models (for a summary cf. Table 1) of our isolated *Drosophila* gut bacteria using the *gapseq* pipeline (Zimmermann et al., 2021). As a last step in the model generation, we used *gapseq's* in-built gap filling algorithm to enable *in silico* growth of the models on the one hand for the ACE/MRS media and on the other hand for the HD medium (see material and methods and Data S1). This additional step takes composition differences of the varying media into consideration. The ACE and MRS media are semi-defined owing to chemically complex components, which makes the *in silico* representation of the growth environment more difficult. We could explain between 73% and 92% of the unknown complex ingredients (yeast extract, peptone, and meat extract) by the help of information from the literature or the respective manufacturer. For HD such problems do not exist, as this medium is chemically completely defined (Piper et al., 2014). The overview of the diet parametrization is provided in Figure S2 as well as Data S2. In the course of generating the models, we took great care to correct for stochiometric inconsistencies, mass and charge imbalances, as well as metabolite connectivity (see materials and methods section and Table 1). All models were tested for model quality using the MEMOTE tool (Lieven et al., 2020) and resulted in at least 77% model scores (see Data S3 and materials and methods).

### *In silico* biomass and signature metabolite production by the different genome-scale metabolic network models

In order to model growth of the different isolated gut bacteria alone as well as in combination, we performed dynamic and agent-based simulations of bacterial population growth and metabolic fluxes using the BacArena software package (Bauer et al., 2017). In brief, BacArena allows growth simulation of single-species population and multi-species microbial communities in a spatially limited compartment, including the calculations of the changing medium composition due to the metabolite utilization and production by individual bacterial cells. Thus, the metabolism of the organisms is calculated in a time-resolved manner with the biomass production as the objective function (for information concerning the biomass production and objective function, please see material and methods as well as Data S1). BacArena provides the metabolic fluxes, growth pattern, and concentrations of the medium for each time point of each individual species present in the *in silico* experiment. This allows the determination of possible cross-feeding and/or physiological interactions in a multi-species *in silico* culture experiment.

As a starting point, we performed single bacteria growth simulations in the three different media MRS, ACE, and HD. An uncertain parameter was the amount of oxygen entering the system. Acetobacteraceae are aerophilic, whereas lactobacilli are microaerophilic and tolerate only a small amount of oxygen. Furthermore, it is still unknown how much oxygen is present in the larval and adult *Drosophila* gut. Given that our goal was to model the situation within the *Drosophila* gut where the two genera would meet each other, we performed all simulations in the presence of 0.1 mM oxygen, which represents a microaerobic situation (Ito et al., 2002).

Of the lactobacilli, the two *L. plantarum* models showed good growth on all media (Figures 1D–1F). *L. brevis*, in contrast, showed only limited biomass production in the MRS, ACE, and HD simulations (Figures 1D–1F). The *A. indonesiensis* and *A. pasteurianus* models all result in strong biomass production in simulations utilizing the ACE and MRS media (Figures 2D and 2E). On the HD, however, all *Acetobacter* strain model simulations only showed low biomass production (Figure 2F). When we compared our *in silico* growth simulation results to the actual wet-lab data (Figure S1), our lactobacilli simulations fitted the experimental data overall better. So far, the reasons for the discrepancies of the *Acetobacter* simulations are not clear. Yet, the appropriate simulation of growth magnitudes is inherently difficult using FBA (see discussion) and might depend on many parameters. For our experiments, however, we focused on the identification of growth dependencies and metabolite exchanges, which are only considering relative changes and are thus unaffected by these shortcomings.

Next, we investigated the production of certain signature metabolites by the different models. Several *Lactobacillus* species are able to use the phosphoketolase pathway and are thus heterolactic (Spector, 2009). On top of the lactobacilli signature metabolite lactate, heterolactic bacteria also produce acetate. Here, we thus tested for a possible heterolactic behavior of our *L. plantarum* and *L. brevis* models. For the *Acetobacter* models, we did not expect such a behavior and only a prominent production of acetate.

**Figure 3:**
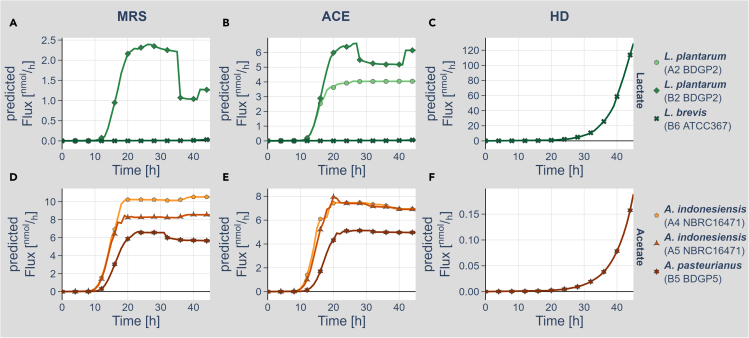
*In silico* production of signature metabolites by the different genome-scale metabolic network models (A–C) Production of lactate by the *L. plantarum* (A2, light green, dot), *L. plantarum* (B2, medium green, check), and *L. brevis* (B6, dark green, cross) genome-scale models on MRS (A), ACE (B), and HD (C) media, respectively. (D–F) Production of acetate by the *A. indonesiensis* (A4, light orange, pentagon), *A. indonesiensis* (A5, medium orange, triangle), and *A. pasteurianus* (B5, dark orange, star) genome-scale models on MRS (D), ACE (E), and HD (F) media, respectively. Please note that not all models produced the respective signature metabolite on the given medium. Metabolite production curves represent mean values of at least 12 simulation runs. An interactive version of the figure is available as Data S4 and detailed model data are available at https://doi.org/10.17632/2tgjd6y4zb.1.

As flux-balance simulations can vary to some extent in terms of individual flux predictions due to stochastic effects, we performed the simulations 100 times to identify the most likely metabolite production behavior (Figure S3). Figures 3 and S4 show representative simulation results (Data S4 is an interactive version of Figure 3, which provides all predicted metabolite productions). Lactate production was mostly limited to *L. plantarum* (B2) on the MRS and ACE diets, *L. plantarum* (A2) on the ACE diet, and *L. brevis* (B6) on the HD (Figures 3 and S3). None of the *Acetobacter* models produced lactate (Figures S3 and S4).

All *Acetobacter* model simulations resulted in prominent acetate production on the ACE and MRS growth media (Figures 3D, 3E and S3). Yet, on the HD only *A. pasteurianus* (B5) was producing acetate (Figures 3F and S3). For the *Lactobacilli*, only the two *L. plantarum* models showed prominent acetate production on the MRS and ACE media (Figures S3 and S4). On the HD, all *Lactobacilli* showed acetate production (Figures S3 and S4). Altogether, our simulations thus reveal a heterolactic behavior of the isolated lactobacilli as well as demonstrate the expected metabolite production for the *Acetobacter* models. Next, we investigated the co-culturing behavior *in silico*.

### Simulating the co-culturing of *Lactobacillus* and *Acetobacter*

**Figure 4:**
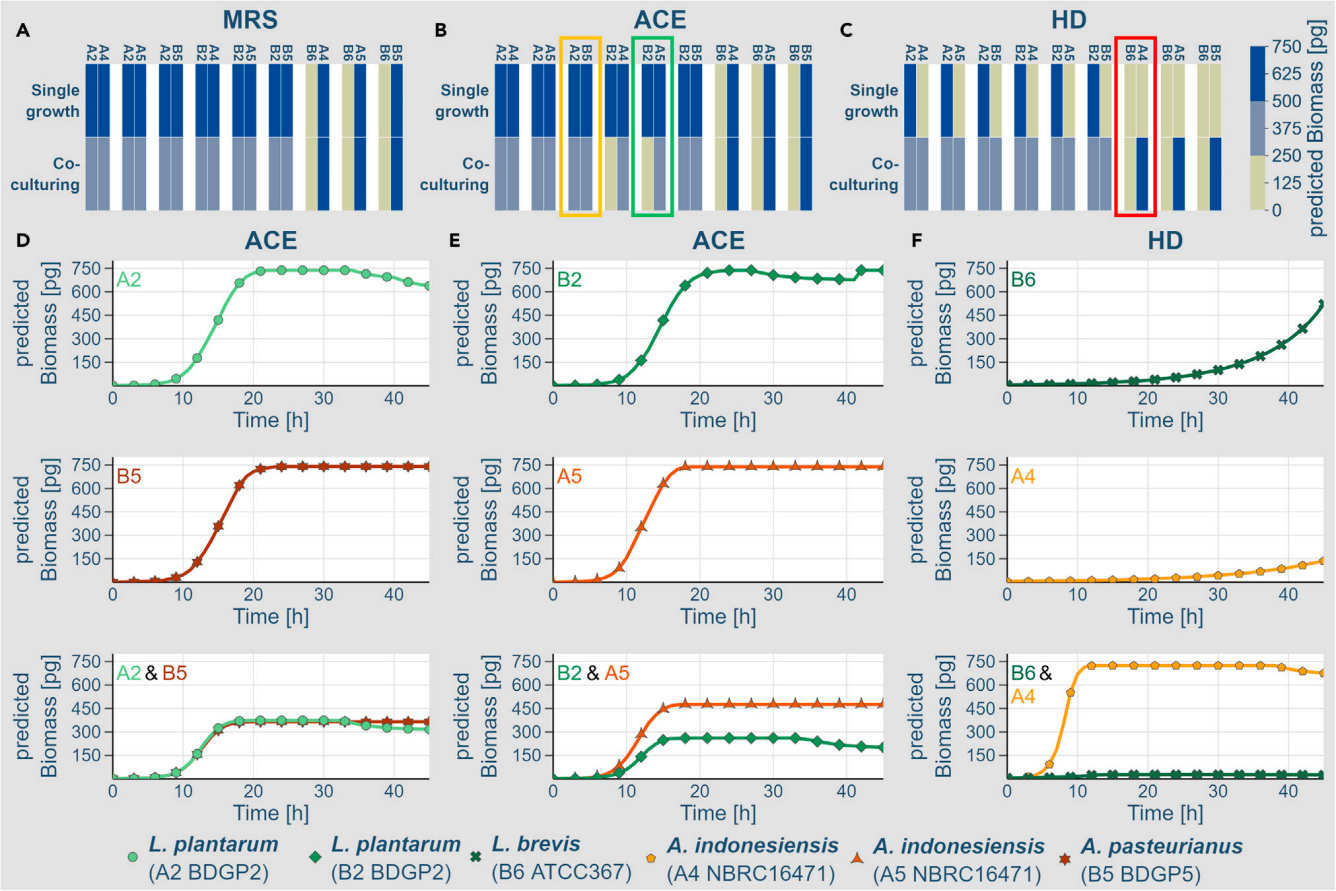
*In silico* co-culturing of *Lactobacillus* and *Acetobacter* (A–C) We simulated the growth of all individual as well as pair-wise combinations of the *Lactobacilli* and *Acetobacter* models on the MRS (A), ACE (B), and HD (C) media. The plots summarize the color-coded biomass produced after 45 h of simulated growth. Total amount of produced biomass from 0–250 pg: beige, equals no or weak growth; 250–500 pg of predicted biomass: light blue; equals intermediate growth, and 500–750 pg of predicted biomass: dark blue; equals strong growth. (D–F) Detailed time-resolved data for three different examples of single organism growth simulations as well as the simulated growth of the combination of the bacteria. D (refers to orange box in B) shows an example of the most trivial growth behavior, where the combination of *L. plantarum* (A2, light green, dot) and *A. pasteurianus* (B5, dark orange, star) on the ACE medium limits the growth of each other based on the impact of space and resource competition. E (relates to green box in B) shows an example of a detrimental outcome of the combination of bacteria. *L. plantarum* (B2, medium green, check) and *A. indonesiensis* (A5, medium orange, triangle) grow individually well on the ACE medium. The combination, however, results in a prominent block of the *Lactobacillus* growth, perhaps due to resource competition effects. F (relates to red box in C) shows a probiotic activity of *L. brevis* (B6, dark green, cross) on the growth of *A. indonesiensis* (A4, light orange, pentagon) on the HD. Both bacteria individually only show minute biomass production on the HD, whereas the combination results in a prominent growth of *A. indonesiensis* (A4, light orange, pentagon).

Our key question was whether bacteria present in the gut could affect each other's growth. For other gut microbiome members of the fly such beneficial metabolite exchange behavior could be recently demonstrated (Consuegra et al., 2020a; Henriques et al., 2020). For the species isolated in this study, we detected prominent growth differences in the different growth media *in vitro* (Figures 1 and 2) as well as *in silico* (Figures 1 and 2). Our hypothesis was that the growth of co-cultures could be different from the growth of pure cultures based on the exchange of metabolites. If one is able to predict the impact of an exchange of metabolites between the different species of a gut microbiome as well as the impact of the metabolite exchange, one could design prebiotics, which means metabolites promoting the growth of a certain beneficial gut microbiome constituent. In order to test for such potential growth-promoting effects, we performed simulations comparing the mono-inoculations to all pair-wise combinations of *Acetobacter* and lactobacilli. In order to quantify potential growth effects, we first estimated the predicted biomass production after 45 h for the individual or co-cultured growth. Figures 4A–4C show the color-coded results for all individual and combined growth conditions on the MRS (A), ACE (B), and HD (C) media (all simulation data are available in the supplement). In Figures 4D–4F we highlight three detailed representative modeling outcomes from the overview representation in Figures 4A–4C (orange box in B relates to D, green box in B relates to E, and red box in C relates to F).

First, we consider the predicted growth curves of singular (upper two panels) or combined (lowest panel) *L. plantarum* (A2) and *A. pasteurianus* (B5) on ACE medium (Figure 4D) as an example of a trivial growth behavior. Both bacteria individually grow very well on the ACE medium. When combined, however, the available space gets limiting and thus both bacteria just reach half of the arbitrarily set maximum possible biomass production of 750 pg. Thus, the two bacteria only affected their mutual growth in terms of a limitation of the available resources. The combination of bacteria, however, can also result in non-trivial growth effects. Simulations with the *L. plantarum* (B2) and *A. indonesiensis* (A5) models on the ACE medium, for example, result individually in very high biomass production (Figure 4E). Yet in combination, the *Acetobacter* model results in higher biomass production, whereas the *Lactobacillus* model results in much lower biomass production (Figure 4E). Thus, the presence of *Acetobacter* apparently limits the biomass production of the *Lactobacillus* model, perhaps by winning the competition about the available resources.

Most striking, however, the combination of *L. brevis* B6 and *A. indonesiensis* A4, which individually produce on the HD only very little biomass in simulations (Figure 4F), results in a surprisingly prominent biomass production of *Acetobacter* (Figure 4F). In fact, the combination of *L. brevis* (B6) and all *Acetobacter* models resulted in such a growth behavior (Figure 4C). Thus, only a small amount of *Lactobacillus* was necessary to allow prominent biomass production of the *Acetobacter* model and *Lactobacillus* serves as a probiotic for *Acetobacter* in our simulations.

### Analysis for metabolites exchanged between *Acetobacter* and *Lactobacillus*

**Figure 5:**
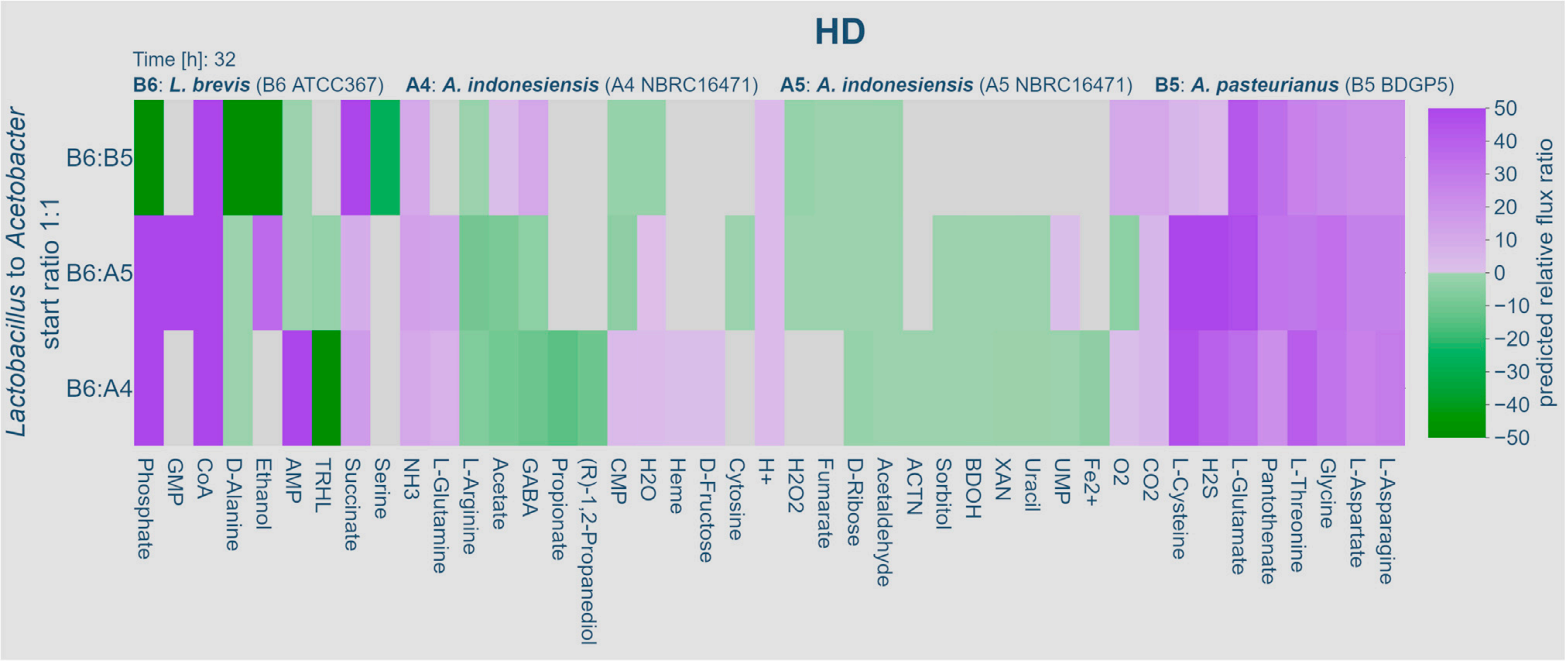
Flux of exchange reactions during the co-culturing of *Acetobacter* and *Lactobacillus* on the HD We simulated the combined growth of *Acetobacter* and *Lactobacillus* on the HD and monitored the respective fluxes of the exchange reactions (thus, the fluxes representing an uptake or excretion of a given metabolite) over time. Exchange reactions are defined as reactions (or passages) where metabolites can flow in and out of the metabolic network and therefore the organism or cell. They can be subjected to different constraints such as diffusion or Michaelis-Menten kinetics of metabolite transporters, but for most reactions, only boundary thresholds can be set as the real-world flux rates are unknown. Further information on exchange reaction is found in Cotten and Reed (2013); Orth et al. (2010). For the sake of simplicity, we combined the individual fluxes into a normalized quotient, where a positive sign represents the same directionality (e.g., both bacteria secrete a given metabolite) of the individual fluxes and a negative sign represents opposite directionalities (e.g., one bacterium secretes a given metabolite and the other consumes it). The heatmap represents the flux ratios at 32 h of growth (an interactive version of the plot for all time points is provided as Data S5). Gray color represents that the respective metabolite is either not present or only transported by one of the two bacteria (not shown in color scale on the right); green color opposite and lilac color same flux directionalities. Multiple metabolites consistently show opposite flux directionalities across bacterial species combinations and across the time line.

The results of our co-occurrence simulations suggest that growth interdependencies between the different gut bacteria exist. Ultimately, the simulations should result in predictions ready to test in *in vivo* experiments. Thus, we concentrated on the following on the growth simulations performed with the HD, as with this defined diet, we can control and fine-tune its constituents. In addition, this diet can also be used in the future to monitor the growth of the bacteria in combination with their natural host *D. melanogaster*. In terms of a probiotic activity of *L. brevis* for *A. indonesiensis* we envisioned that the *Lactobacillus* either removed a growth-inhibiting or secreted a growth-promoting factor thus enabling *Acetobacter* to produce biomass in our simulations. Thus, we monitored the excretion and uptake rates of both bacteria over time within the simulations. For an easier detection of a net efflux or uptake, we formed a quotient between the individual uptake rates and normalized the values (see materials and methods). This allowed us to plot the exchange reactions in a heatmap (Figure 5) where a positive value means that both bacteria take up or excrete the given metabolite and a negative value means that the bacteria show a reciprocal metabolite transport behavior. Thus, a negative value is consistent with the excretion of a given metabolite from one bacterium and the uptake of the same metabolite by the other species. Figure 5 shows the situation after 32 h of growth (see Data S5 for an interactive version of the figure providing the data for all time points). Many transport reactions had a positive sign, and thus the direction of the transport pointed in the same direction in both bacteria. Few reactions, however, consistently showed a negative sign, which is in line with an exchange of the given metabolite. Among those, D-Alanine, L-Arginine, D-Ribose, Acetaldehyde, Fumarate, and Butane-2,3-diol (BDOH) showed the most prominent exchange behavior.

### Growth-promoting effect of singular metabolites added to *Acetobacter* cultures

**Figure 6:**
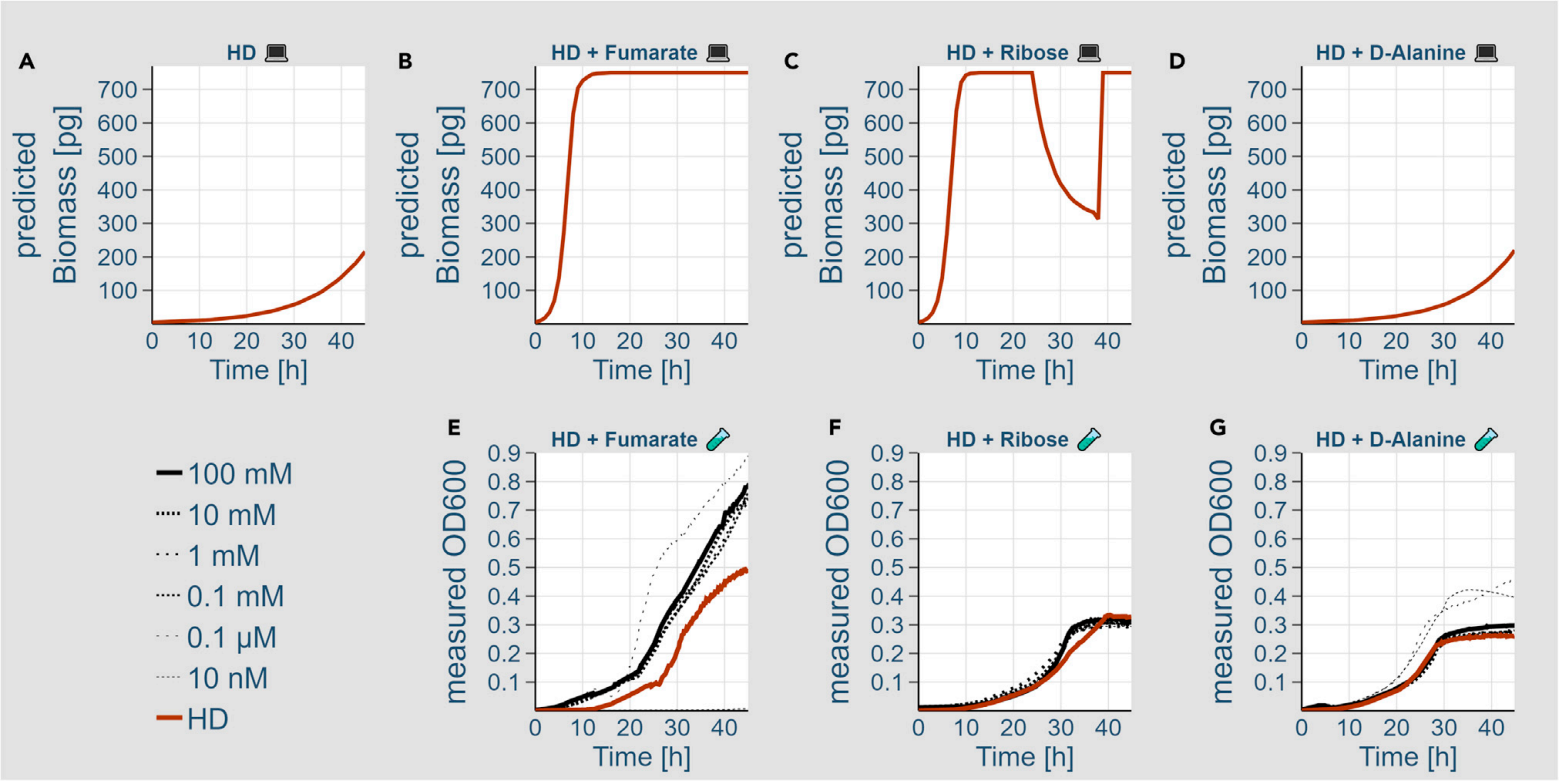
Growth-promoting effect of singular added metabolites (A–D) *In silico* biomass production of *A. pasteurianus* (B5) on the standard HD. *In silico* biomass production of *A. pasteurianus* (B5) on HD with 10 mM (B) Fumarate, (C) Ribose, and (D) D-Alanine. (E–G) Actual growth measurements of *A. pasteurianus* (B5) on HD (dark orange) with Fumarate (E), Ribose (F), or D-Alanine (G) (10 nM, 0.1 μM, 0.1 mM, 1 mM, and 100 mM; black color and different dashed lines). *In silico* experiments (A, B, C, and D) are represented by the computer, whereas the wet-lab experiments (E, F, and G) are represented by the small reaction tube pictograms.

We tested next whether the addition of any of the metabolites shown in Figure 5 to the HD growth medium simulations is sufficient to improve the growth of *A. indonesiensis*, which alone showed only poor biomass production on the HD medium (Figure 6A). Of the 43 metabolites tested (Figure S5), only 10 metabolites showed a growth-promoting effect *in silico*. Those were indeed enriched for the metabolites, which showed a predicted exchange from one bacterial species to the other (negative sign in Figure 5). The *in silico* addition of the TCA intermediate fumarate, for example, resulted in prominently increased predicted biomass production (Figure 6B). The same growth-promoting effect is visible in the *in silico* prediction of D-Ribose added to the HD medium (Figure 6C). No growth-promoting effect was visible when D-Alanine was added to the HD medium in the *in silico* prediction of *A. pasteurianus* B5 (Figure 6D), whereas biomass production of *A. indonesiensis* A4 and A5 was promoted (Figure S5). Thus, the simulations suggested that already the exchange of a singular metabolite between the bacterial species could result in a growth-promoting effect.

Finally, we tested for the experimental validation of the predicted growth-promoting effects. For this purpose, we recorded growth curves of *A. pasteurianus* (B5) in HD containing varying concentrations of fumarate (Figure 6E), D-Ribose (Figure 6F), and D-Alanine (Figure 6G). With fumarate and D-Ribose, we selected metabolites that showed *in silico* a prominent growth-promoting effect on all *Acetobacter* species (Figure S5), whereas D-Alanine did not result in a full growth rescue of *A. pasteurianus* (B5), but only the other two *Acetobacter* species (Figure S5). D-Ribose alone was not sufficient to improve the growth of *A. pasteurianus* (B5) prominently (Figure 6F). Yet, the addition of fumarate and D-Alanine in different concentrations showed a prominent positive effect on the growth of the bacteria (Figures 6E and 6G).

Altogether, our results suggest that microbiome members are metabolically connected, thus affecting the growth of individual microbiome members. The strategy presented herein consisting of the isolation of distinct bacteria, their genome sequencing, and subsequent *in silico* modeling of growth and metabolism thus proved successful to identify metabolite exchange and growth-promoting metabolites. Future experiments targeted to investigate combinatorial effects of metabolite additions as well as the contribution of the hosts' metabolism will further extend our understanding of the complex interplay among the gut microbiome members.

## Discussion

In this study, we analyzed multiple members of the *Drosophila* gut microbiome by a combination of *in vitro* and *in silico* experiments. In total, we isolated six bacterial strains from laboratory-reared *Drosophila* flies followed by *in vitro* growth experiments, resequencing, and genome assembly and *in silico* growth and metabolism modeling analyses.

First, we tested for a biomass production of the singular bacteria models on ACE, MRS, and HD. *L. plantarum* was able to generate high amounts of biomass on the ACE medium, whereas *L. brevis* was not (Figures 1D–1F). Similar growth was detected on the MRS medium and on HD. All *Acetobacter* models resulted in high biomass production on the ACE and MRS media and only very low biomass production on the HD (Figures 2D–2F). Our models mostly recapitulated the corresponding actual growth experiments (Figures 1, 2, and S1). Especially the poor growth of the *L. brevis* isolate was detected *in vitro* and *in silico* (Figures 1, 2, and S1). The reason for this growth deficit is to date not clear. For some of the organisms, such as *A. indonesiensis* on the ACE medium, the modeling results deviate from the actual measurements in terms of the magnitude of the effect (Figure S1). This is a problem seen in many modeling approaches, which might be based on a variety and most likely a combination of many parameters, including gaps in the model, confounding factors, and the lack of certain environmental conditions in the modeling procedure. Furthermore, the modeling procedure depends on the requirement to define “exchange reactions,” which are thresholds setting the boundaries for metabolic fluxes going into and out of the model. Although these thresholds can be controlled by different constraints such as diffusion or Michaelis-Menten kinetics of metabolite transporters (Cotten and Reed, 2013; Orth et al., 2010), for most of the reactions, these boundaries are not experimentally validated and thus the model itself is largely underdetermined. Furthermore, also the biology of the given bacterium might be a cause for deviations between the experimental and modeling data. A prerequisite of the FBA procedure is the assumption that an objective function is optimized in terms of a maximization. Often as well as in our study the optimized objective function is biomass production. Previous studies, however, demonstrated that several microorganisms operate at a sub-maximal growth rate (Fischer and Sauer, 2005; Schuetz et al., 2007, 2012). The reasons for this behavior are not yet always clear.

Altogether, these parameter variations and modeling uncertainties will result not only in deviations of the magnitude of, e.g., biomass production, but also in kinetic differences, e.g., in terms of the growth rate. With variations in the build-up of biomass, also the mass transfer will vary, thus potentially resulting in more prominent differences between the computed and wet-lab results. Important, these confounding characteristics of the modeling procedure apply to the single and the multiple species growth simulations. The latter, however, will of course be even more severely affected by differences in the growth rates of the individual species that make up the consortium as the mass ratios between the species will also affect the mass transfer of metabolites. Furthermore, also the details concerning the juxtaposition (directly neighbored versus located in, e.g., different compartments of the gut) as well as variations in the initial mass ratio, thus the relative abundance of each species, will prominently affect the individual growth rates and mass transfer. Further experimental data including, e.g., localization studies, measurements of the individual abundance of bacterial species, and metabolic labeling experiments to determine flux rates as well as refinements of the models will help to improve the modeling outcome in the future.

Future iterations and refinements of the models will also need to target the optimization of the growth condition parameterization. Our simulations using the HD medium is a first step in the direction of modeling the actual growth conditions within the fly gut, as all bacteria as well as the host can thrive on this medium. The standard diet most often used to rear *Drosophila* is complex and undefined, often containing live or dry yeast, molasses, or treacle, which makes the parameterization and modeling very complex. Also, the exact conditions within the gut are still not clear as, e.g., metabolite concentrations might vary along the anterior-posterior axis of the gut as well as across the diameter of the gut. Thus, further experimental and modeling work will be needed to decipher these details in the future.

On top of testing the biomass production, model validation also included the analysis of expected signature metabolite production. *Acetobacter*, for example, is known to oxidize sugars or ethanol to acetic acid (Raspor and Goranovič, 2008), whereas lactobacilli produce glucose-derived lactic acid as the main product (Hatti-Kaul et al., 2018). Both metabolic models were able to recapitulate this behavior (Figure 3). It is intriguing that the previously described heterolactic metabolism of lactobacilli (Spector, 2009) could also be recapitulated for our isolated bacteria (Figure S3) suggesting that our models result in realistic metabolic behavior predictions. Of note, however, some of the predictions need to be considered with care. Our simulations, for example, also revealed the production of H_2_O_2_ and also H_2_S. Both substances can act as inhibitors of bacterial growth, especially in higher concentrations (Alt et al., 1999; Reis et al., 1992). Nevertheless, some Acetobacter species were demonstrated to produce H_2_S under certain conditions (Ahmad et al., 2004). Thus, so far it is not clear whether the neutral or even positive effect of the presence of these substances on the growth (Figure S5) is real or based on the limitation of FBA to predict correctly growth-inhibiting and detrimental effects of certain metabolites.

The main goal of our study was to test whether we can predict metabolic growth-promoting inter-species interactions. If possible, this could open up the door to design tailored prebiotics to promote or hinder the growth of certain gut microbiome members. For our simulations, we tested all pair-wise combinations of *Acetobacter* and *Lactobacillus* on the three different media ACE, MRS, and HD. Many combinations were neutral in a way that the growth of the singular bacteria was similar or identical in the singular and combination situation (Figures 4A–4C; the complete dataset is provided in the supplement). In case both bacteria showed high growth in single growth simulations, the combination resulted in a competitive situation, which caused both bacteria to grow less (e.g., Figure 4D). On top of these trivial situations, however, we also observed inhibitory and stimulatory interactions. The *L. plantarum* strain B2 and *A. indonesiensis* strain A5 result in comparable and high biomass production in the ACE medium when grown independently (Figure 4E). The combination, however, does not result in an equal reduction of the biomass to an intermediate level, but in contrast to a much stronger reduction of the *Lactobacillus* biomass production, whereas *Acetobacter* production got increased (Figure 4E). Likely, this effect is based on resource competition, which might also play a role within the gut of the *Drosophila* host. Even more astonishing was the stimulatory effect of combining the individually poor biomass producers *L. brevis* and either of the *Acetobacter* models, which we were able to track down to the exchange of selected metabolites (Figures 4F and S5). For fumarate and D-Alanine, we already were able to confirm the growth-promoting effect by simply adding these metabolites to the HD medium (Figure 6). Ribose, however, did not result in the expected growth rescue. At this point, the reasons for this discrepancy are unclear. Whether additional metabolites could also rescue the growth deficit to a similar extent is at this point unknown. Similarly, it is also not clear how the co-culturing of the organisms in the end affects each other as beneficial and competition effects most likely will play a role and thus a more complex growth effect will arise.

Fumarate and D-Alanine could affect the growth of the bacteria by different means. Thus, we considered different possibilities and cross-validated these using our modeling data. Formally, the metabolites could complement auxotrophies. Based on our modeling and experimental data, however, we exclude this possibility, as the bacteria also grow without the supplementation in the MRS or ACE media (Figures 1 and 2). Furthermore, the compounds could function as additional C- or N-source and enter the metabolism. Fumarate indeed is a central metabolite of the TCA. Thus, its uptake could enhance the overall capacity of the TCA. Various TCA intermediates further serve the biosynthesis of different amino acids, which potentially could also benefit biomass production. For *Acetobacter pomorum* a potential use of fumarate by the enzyme succinate dehydrogenase (EC1.3.5.1, present in TCA) was discussed where fumarate serves as an O-donor for the production of NAD+ and NADP+ from Aspartate (Consuegra et al., 2020b). D-Alanine, in contrast, could be converted first to L-Alanine and subsequently to pyruvate, which serves as a carbon and energy source. When we analyzed the corresponding flux differences of the modeling performed in the presence or absence of the metabolites in the HD (Figure S6 and Data S6 and S7), we indeed detected a number of corresponding flux changes. First, we consider the situation where D-Alanine was added to the HD. Here, we see an increase in the flux associated with the conversion of D-Alanine to Pyruvate, as expected (Figure S6A). Pyruvate production is further enhanced coming from oxaloglutarate (Figure S6B). Other prominent changes include the change of direction of the fluxes from fumarate to malate and oxaloacetate (Figures S6C and S6D), the production of isocitrate from citrate (Figure S6E), the production of S-Succinyl-dihydrolipoamide from oxaloglutarate (Figure S6F), or the enhanced production of glutamate-derived amino acids such as glutamine (Figure S6G). Many of these enzymatic reactions are also affected by adding fumarate to the HD. Overall, the fumarate-induced flux changes of the TCA reactions are, however, bigger as from D-Alanine. Fumarate also resulted in a third possibility to enhance the pyruvate production coming from oxaloacetate (Figure S6H). The fumarate addition induced higher flux changes, which might provide an explanation for the overall bigger growth rescue phenotype detected in the actual growth experiments (Figure 6E). A recent report also targeted the prediction of *Drosophila* gut microbiome metabolite interactions using *in silico* models (Ankrah et al., 2021). The authors independently also revealed that TCA intermediate metabolites appear to be prominently exchanged between gut microbiome members. In their simulations, the authors used different media than we did, but still found a similar range of exchanged metabolites. Reassuringly, many of the exchanged metabolites are shared by our and the published study. In our extended studies, however, we did not detect a prominent growth-promoting effect for some of these in our simulations (e.g., acetate, succinate, different individual amino acids). Yet, several metabolites detected in both studies (e.g., acetoin, acetaldehyde) clearly resulted in an individual growth-promoting activity (c.f. Figure S5 and Ankrah et al., 2021).

Our results support the possibility to use genome-scale models in combination with agent-based growth simulations to predict meaningful microbiome cooperativity. In the future, extending this approach to additional microbiome constituents and/or the metabolism of the host *D. melanogaster* will be exciting and perhaps pave the way to analyze also the much more complex microbiomes of higher organisms.

### Limitations of the study

There are limitations in the modeling of growth-promoting bacterial metabolic interactions. On the one hand, this is true for the modeling side as outlined above. For example, FBA assumes optimization and maximization of a given parameter such as biomass production, yet organisms sometimes operate at a sub-optimal level. Furthermore, our knowledge of many parameters required for the modeling such as nutrient distribution along the gut, nutrient uptake rates, and transport reaction efficacies are unknown, which results in the necessity to make assumptions that are in the best case imprecise and in the worst case wrong. Further iterations and improvements on the modeling and experimental side might solve some of these shortcomings using, e.g., isotope labeling experiments. On the other hand, uncertainties concerning the biology exist. For example, we used laboratory-reared flies and detected the most prominent microbiome growth interactions on a minimal diet used for the growth of *Drosophila*. In the future, bacteria from wild-reared animals grown under natural conditions should be used, which, however, will be experimentally very challenging. Finally, our analyses were performed with simple consortia. Ultimately, complex mixtures with varying relative microbial species abundancies and consisting of more species will be required to estimate the true importance of metabolic cross-feeding phenomena among gut microbiota.

## STAR★Methods

### Key resources table

**Table:** 

REAGENT or RESOURCE	SOURCE	IDENTIFIER
**Bacterial and virus strains**		

*Acetobacter pasteurianus*	This paper	B5
*Acetobacter indonesiensis*	This paper	A4
*Acetobacter indonesiensis*	This paper	A5
*Lactobacillus plantarum*	This paper	A2
*Lactobacillus plantarum*	This paper	B2
*Lactobacillus brevis*	This paper	B6

**Chemicals, peptides, and recombinant proteins**		

L-arginine HCl	Sigma-Aldrich	Cat#A5131
L-alanine	Sigma-Aldrich	Cat#A7627
L-asparagine	Sigma-Aldrich	Cat#A0884
L-aspartic acid	Sigma-Aldrich	Cat#A6683
L-cysteine	Sigma-Aldrich	Cat#C1276
L-glutamic acid monosodium salt monohydrate	Sigma-Aldrich	Cat#G5889
L-glutamine	Sigma-Aldrich	Cat#G3126
Glycine	Sigma-Aldrich	Cat#G7126
L-histidine	Sigma-Aldrich	Cat#H8000
L-isoleucine	Carbolution	Cat#CC10025
L-leucine	Sigma-Aldrich	Cat#L8912
L-lysine HCl	Sigma-Aldrich	Cat#L5626
L-methionine	Sigma-Aldrich	Cat#M9625
L-phenylalanine	Sigma-Aldrich	Cat#P2126
L-proline	Sigma-Aldrich	Cat#P0380
L-serine	Sigma-Aldrich	Cat#S4500
L-threonine	Carl Roth	Cat#T206
L-tryptophan	Sigma-Aldrich	Cat#T0254
L-tyrosine	Sigma-Aldrich	Cat#T3754
L-valine	Sigma-Aldrich	Cat#V0500
Sucrose	Carl Roth	Cat#4661
Cholesterol	Sigma-Aldrich	Cat#C8667
choline chloride	Sigma-Aldrich	Cat#C1879
myo-inositol	Sigma-Aldrich	Cat#I7508
Inosine	Sigma-Aldrich	Cat#I4125
Uridine	Sigma-Aldrich	Cat#U3750
Tween20	Sigma-Aldrich	Cat#P7949
KH2PO4	Grüssing Gmbh	Cat#120171000
NaHCO3	AppliChem	Cat#AP131638
CaCl2.6H2O	Sigma-Aldrich	Cat#442909
CuSO4.5H2O	AcrosOrganics	Cat#A0302205
FeSO4.7H2O	Sigma-Aldrich	Cat#F7002
MgSO4.7H2O	AppliChem	Cat#A6287
MnCl2.4H2O	Sigma-Aldrich	Cat#M3634
ZnSO4.7H2O	Sigma-Aldrich	Cat#Z0251
thiamine (aneurin)	Sigma-Aldrich	Cat#T4625
Riboflavin	Sigma-Aldrich	Cat#R4500
nicotinic acid	Sigma-Aldrich	Cat#N4126
Ca pantothenate	Sigma-Aldrich	Cat#P21210
pyridoxine-HCL	Sigma-Aldrich	Cat#P9755
Biotin	Sigma-Aldrich	Cat#B4501
folic acid	Sigma-Aldrich	Cat#F7876
HPLC	Fisher Scientific	Cat#231-791-2
Fumarate	BLD Pharmatech Gmbh	Cat#BD131629
D(-)-Ribose	AcrosOrganics	Cat#10320164
D-Alanine	Carbolution	Cat#CC10041
Acetic acid glacial	VWR Chemicals	Cat#KRAF20104
Glucose	Fisher Scientific	Cat#10529190
Sodium acetate	Grüssing Gmbh	Cat#121111000
Cycloheximide	AppliChem	Cat#A0879
Peptone	Carl Roth	Cat#8986.2
Yeast Extract	BD Company	Cat#212750
Beef Extract	Carl Roth	Cat#X975
Triammonium citrate	Sigma-Aldrich	Cat#A1332
Tween20	Sigma-Aldrich	Cat#P7949
Ethanol	Honeywell	Cat#32221
MRS agar plates	Thermo Scientific	Cat#CM0361B
Proteinase K	Thermo Scientific	Cat#AM2546
Lysozyme	Sigma-Aldrich	Cat#34046
Phusion HF Polymerase	New England Biolabs	Cat#M0530
Tris-HCL	Roche	Cat#10812846001
EDTA	AppliChem	Cat#1.08452
Triton™ X-100	Sigma-Aldrich	Cat#X100
Bleach	DanKlorix Hygiene Reiniger	N/A
Agar	Becton Dickinson	Cat# 10455513
Polenta	Verival; Pronurel Bio	N/A
Soy flour	Bauck Hof	N/A
Yeast	Bruggeman	N/A
Treacle	Original Grafschafter Goldsaft	N/A
Malt extract	Demeter	N/A
Nipagin	Sigma-Aldrich	Cat# H3647
Propionic acid	Acros Organics	Cat#AC149300010
Tween80	Sigma-Aldrich	Cat#P1754

**Critical commercial assays**		

TOPO TA Cloning Kit for Sequencing	Invitrogen	Cat#K4575J10
QIAamp DNA Mini Kit	Qiagen	Cat#51304

**Deposited data**		

Raw and analyzed data	This paper	DOI: 10.17632/2tgjd6y4zb.1

**Experimental models: organisms/strains**		

*D. melanogaster* wildtype strain Oregon-R	Bloomington Drosophila Stock Center	BDSC: 5; FlyBase: FBsn0000276
*D. melanogaster* white[1118]	Vienna Drosophila Resource Center	VDRC:60000

**Oligonucleotides**		

GM3F: AGAGTTTGATCMTGGC	Klindworth et al., 2013	N/A
GM4R: TACCTTGTTACGACTT	Klindworth et al., 2013	N/A

**Software and algorithms**		

Python 3.8	Python Software Foundation	https://www.python.org
R Studio 1.2.5042	RStudio, Inc.	https://www.rstudio.com
R 3.6.1	R Foundation for Statistical Computing	https://www.R-project.org
BacArena 1.8	Bauer et al., 2017	https://bacarena.github.io
gapseq 1.1	Zimmermann et al., 2021	https://github.com/jotech/gapseq
Plotly 4.14.3	Plotly Technologies Inc.	https://plot.ly
Code to re-perform analyses and to recapitulate the plotting.	This paper	https://gitlab.com/Beller-Lab

### Resource availability

#### Lead contact

Further requests for resources should be directed to and will be fulfilled by the lead contact, Mathias Beller (mathias.beller@hhu.de).

#### Materials availability

This study did not generate new materials.

### Experimental model and subject details

#### Fly strains and rearing

The fly lines that were used in this study are *w*^*1118*^ (*white[-]*) and Oregon-R. Flies were maintained at 25°C with 60–70% humidity and a 12 h light/dark cycle. Standard diet contains 0.5% agar (Becton Dickinson), 7.1% polenta (Verival, Pronurel Bio), 0.95% soy flour (Bauck Hof), 1.68% yeast (Bruggeman), 4% treacle (Original Grafschafter Goldsaft), 4.5% malt extract (Demeter). All diets contained 0.15% nipagin (Sigma-Aldrich) and 0.45% propionic acid (Acros Organics).

#### Isolation of bacterial species from Drosophila

In order to analyze different bacterial species from the gut microbiome of *Drosophila*, both *white[-]* and Oregon-R male flies (9 individuals) were surface sterilized by washing with 10% bleach, 70% ethanol and PBS before homogenization and plating on MRS and ACE agar plates. MRS agar plates (Oxoid, Thermo Scientific) contain (in 1000 mL dH_2_O): Agar (15 g), casein peptone, tryptic digest (10 g), meat extract (10 g), yeast extract (5 g), glucose (20 g), Tween 80 (1 g), K_2_HPO_4_ (2 g), Na-acetate (5 g), (NH_4_)_2_ citrate (2 g), MgSO_4_ x 7 H_2_O (0.2 g), MnSO_4_ x H_2_O (0.05 g), pH 6.2–6.5. ACE agar plates (Blum et al., 2013) contain: (in 1000 mL dH_2_O): Agar (15 g), yeast extract (8 g), casein peptone (15 g), glucose (10 g), after autoclaving: acetic acid (3 mL), ethanol (p.a.) (5 mL) and Cycloheximid (100 mg). The plates were incubated at 28°C for three to five days and single colonies were picked and isolated on new agar plates for three rounds to obtain pure cultures. These were then stored in glycerol stocks for later DNA extraction and analysis.

### Method details

#### Single colony PCR and analysis of 16S rRNA genes

Of the different pure cultures single colonies were picked and transferred into PBS buffer containing 200 μg/ml Proteinase K and 10 mg/ml Lysozyme and incubated for 30 min at 37°C and 2 min at 95°C. The samples were centrifuged for 2 min at 13.000 rpm and the supernatant transferred to a new vial. The 16S rRNA Gen was amplified using the GM3F and GM4R primers (Klindworth et al., 2013) using the Phusion Polymerase (New England Biolabs) which produced a product of about 1500 bp. These PCR products were then ligated into the TOP TA Vector (TOPO TA Cloning Kit for Sequencing, Invitrogen) and transformed into chemocompetent *E. coli* DH5alpha according to the manufacturer's instructions. The vector including the insert was extracted from *E. coli* and the insert analyzed by Sanger sequencing (MWG Biotech). The DNA sequence was afterwards subjected to BLAST analysis to identify the isolated bacterial species.

#### DNA extraction from bacterial species for genome sequencing

The DNA extraction was performed using the Qiagen *QiaAmp DNA Mini* kit according to the manufacturer's recommendation, with the following modifications. Briefly, an inoculation loop was used to pick bacterial colonies from the pure cultures grown on ACE or MRS agar plates and the bacteria were resuspended in gram-positive lysis buffer (20 mg/ml lysozyme; 20 mM Tris·HCl, pH 8.0; 2 mM EDTA; 1.2% Triton®). The following lysis and purification steps were performed according to the kit's protocol for DNA extraction from gram-positive bacteria.

#### Liquid media bacteria growth experiments

For the bacterial growth experiment, we prepared pre-cultures in the respective semi-selective medium (MRS for *Lactobacillus sp.* and ACE for *Acetobacter sp.* (Blum et al., 2013)). Subsequently, we either directly used the optical dense overnight culture or adjusted it to an OD600 of 0.8. Next, we performed a 1:1000 (MRS and ACE) or 1:100 (HD) dilution and distributed the bacteria to transparent 96-well flat bottom plates (Sarstedt). The medium was covered with mineral oil and incubated in a BioTek Synergy Mx Plate Reader with (*Acetobacter*) or without (*Lactobacillus*) shaking for at least 48 hours. Optical density was measured every five minutes. Per experiment, all growth curves were measured in at least triplicate and the figures provide mean values.

#### Whole genome sequencing of isolated bacterial species

The isolated genomic DNA samples from the gut microbiota species were sequenced using the Illumina MiSeq platform following standard procedures. The library preparations and sequencing were performed by the Genomics and Transcriptomics Lab at the HHU.

#### Genome reassembly

For the genome reassembly the tool ASA³P (Schwengers et al., 2020) was used. ASA³P is an automatic, scalable assembly, annotation, and analysis pipeline for genomes of bacterial origin. The pipeline consists of four steps: Processing, characterization, comparative genomics, and reporting. Each step provides different analysis information about the used sequenced genome through different software tools and databases. While processing and reporting is mandatory, the steps of characterization and comparative genomics is optional and can be skipped by the user. The first step processing includes the task of quality control, genome assembly, scaffolding and annotation. The second step of characterization determines the taxonomy, performs a multi locus sequence typing (MLST) analysis, tries to detect antibiotic resistances (ABRs), a detection of virulence factors (VFs), performs a mapping by using quality clipped reads onto reference genomes provided by the user, and annotates single-nucleotide polymorphisms (SNPs). The third step of comparative genomics consists of the calculation of a phylogenetic tree and of a core, accessory and pan-genome while detecting isolate genes. The last step is a graphical presentation of the pipeline results. All ASA³P results are provided in the supplement.

#### Reconstruction of bacterial metabolic models

The sequenced genomes were used to reconstruct their genome-scale metabolic models using the *gapseq* analysis pipeline (Zimmermann et al., 2021). We used for the reconstruction and gap-filling step the MRS, ACE and HD as the growth medium. All metabolic models were created combining each genome sequence and every single medium. During the model generation process, we considered in particular stochiometric consistency, mass and charge balance as well as metabolite connectivity and introduced necessary changes following manual curation. In order to test for the quality of our models, we used the MEMOTE analysis pipeline (Lieven et al., 2020). All analysis results are provided as supplemental data. In brief, the models resulted in at least 77% model quality scores. Most importantly, the key requirements for the models all reached at least 99%. The score was only decreased by e.g. missing gene or metabolite annotation cross-references, which we do not focus on in the present manuscript and have no influence on flux predictions in constraint-based modeling. A central part of genome-scale metabolic models is the biomass reaction, which represents the metabolite consumption for the formation of all cell constituents. The biomass reaction is commonly, and also in this study, used as objective function for flux balance analysis (FBA) or FBA-derived simulation techniques. The gapseq software automatically adds a biomass reaction to the models based on the organism's Gram-staining phenotype in order to account for biomass composition differences due to differences in the structural characteristics of the cell wall. The exact biomass reaction stoichiometries in gapseq are directly derived from ModelSEED (Henry et al., 2010), which in turn derived the biomass reaction definitions from curated genome-scale metabolic models from *Escherichia coli* (Orth et al., 2011) as a proxy for Gram-negative bacteria and *Bacillus subtilis* (Oh et al., 2007) as a proxy for Gram-positive bacteria. The biomass compositions for all *Lactobacilli* models (Gram-positive) and *Acetobacter* models (Gram-negative) are provided in Data S1.

#### Constraint-based modeling

Flux balance analysis (FBA; (Orth et al., 2010)) was used to perform the growth and metabolic flux analysis. The mono- and co-culturing in silico experiments were performed using the BacArena tool (Bauer et al., 2017), which is also based on FBA.

#### In silico growth media

*In silico* experiments used parametrized versions of the experimentally used MRS, ACE and HD media (Supp. Table 1). MRS and ACE medium are semi-defined as the contain complex ingredients such as yeast extract. Therefore, we obtained compositional information from the suppliers of the respective media ingredients (see Data S2). For some media components, which are required to run the simulations, no quantitative information could be obtained. Those compounds were manually curated and added. We limited the number of such manually added compounds to the absolute minimum and provide all media information as supplemental data. The parametrized HD medium is based on the protocol of (Piper et al., 2014), which is completely synthetic and thus did not require any modifications.

#### Calculation of predicted relative flux ratios

To identify reactions with a higher flux and reactions corresponding to a crosstalk between *Lactobacillus brevis* B6 and the *Acetobacter sp.* we calculated a predicted relative flux ratio for each reaction and time point.

We calculated the predicted relative flux ratio as followed:

(Equation 1) $$v_{Ratio,\;Reaction\;i,t}=\frac{v_{Lactobacillus\;B6,\;Reaction\;i,t\;}}{v_{Acetobacter,\;Reaction\;i,t\;}}$$

where $v_{Lactobacillus\;B6,\;Reaction\;i,t\;}$ is the flux of the ${\text{reaction}}_{i}$ of *Lactobacillus* B6 at time point *t* in $\frac{mmol}{gDW*h}$, $v_{Acetobacter,\;Reaction\;i,t\;}$ is the flux of the ${\text{reaction}}_{i}$ of *Acetobacter sp.* at time point *t* in $\frac{mmol}{gDW*h}$.

If the predicted relative flux ratio value is between 1 and -1 we calculated the values as followed:

(Equation 2) $$v_{Ratio,\;Reaction\;i,t}=\frac{1}{v_{Ratio,\;Reaction\;i,t}}\;;1>\;v_{Ratio,\;Reaction\;i,t}>\;-1$$

where $v_{Ratio,\;Reaction\;i,t}$ is the unitless predicted relative flux ratio. We choose this representation of the value range between 1 and -1 to highlight the higher flux value between *Lactobacillus* B6 and the *Acetobacter sp.*

#### Calculation of cumulative flux values

In order to analyse the metabolic impact of an additional metabolite in the holidic diet towards the bacteria grown on the media we calculated the cumulative flux for each time point.

First, we calculated the sum of flux values:

(Equation 3) $$v_{Sum,M,\;Reaction\;i}=\overset{n}{\underset{t=0}{\sum }}v_{M,\;Reaction\;i\;}$$

where $v_{Sum,M,\;Reaction\;i}$ is the sum of flux values over the time *t* with medium M in $\frac{mmol}{gDW*h}$, $v_{M,\;Reaction\;i\;}$ is the flux value at a time point with medium *M* in $\frac{mmol}{gDW*h}$.

Next, we calculated the difference of the sum flux values between the standard holidic diet HD and the medium *M*:

(Equation 4) $$v_{cflux,\;Reaction\;i}=v_{Sum,HD,\;Reaction\;i}-\;v_{Sum,M,\;Reaction\;i}$$

where $v_{cflux,\;Reaction\;i}$ is the difference between the summed flux values of HD and medium *M* over the time *t*.

Finally, we calculated the cumulative flux as followed:

(Equation 5) $$v_{cflux,Reacton\;i}=log\left(\left|v_{cflux,\;Reaction\;i}\right|+1\right)$$

where $v_{cflux,Reaction\;i}$ is the cumulative flux value between HD and the medium *M* for a reaction in $log\left(\frac{mmol}{gDW*h}\right)$. The cumulative flux value can also be calculated for a group of reactions.

### Quantification and statistical analysis

Figures represent averaged or representative results of multiple independent experiments or simulations. The figure legends provide details concerning the N of experiments or simulations. Analyses and Plots were performed with custom Python scripts.

### Additional resources

All data is available at data.mendeley.com under the URL https://doi.org/10.17632/2tgjd6y4zb.1.

## Data Availability

• Genome resequencing data, the genome-scale metabolic networks and bacterial growth data, as well as all data required to reproduce the figures are deposited at Mendeley Data and is available as of the date of publication at https://doi.org/10.17632/2tgjd6y4zb.1. • All original code was additionally deposited at our GitLab account and can be accessed via https://gitlab.com/Beller-Lab. • For any additional questions or information please contact the lead contact.

## Supplemental information

Document S1. Figures S1–S6

Data S1. Detail of the gapseq gap-filling procedure. For details of the procedure please refer to (Zimmermann et al., 2021), related to Table 1

Data S2. Details of the media composition and the data sources used for the parameterization, related to Table 1 and Figures 1ff

Data S3. MEMOTE analysis results for the quality control measurements of the genome-scale model quality. The zip folder contains all individual results as PDF files as well as interactive versions, which can be accessed via the html files, related to Table 1

Data S4. Metabolite production of singular bacteria *in silico* growth on the three different growth media (interactive version of Figure 3 of the main text), related to Figure 3

Data S5. Metabolite flux during co-culturing of *L. brevis* (B6) and the three *Acetobacter* sp. models on the HD (interactive version of Figure 5 of the main text), related to Figure 5

Data S6. Flux differences of *A. pasteurianus* (B5) grown on HD or HD with D-Alanine addition, related to Figure 6

Data S7. Flux differences of *A. pasteurianus* (B5) grown on HD or HD with Fumarate addition, related to Figure 6
