# Supplementary material for: Modeling Drosophila gut microbe interactions reveals metabolic interconnectivity
Source: iScience. 2021 Oct 6;24(11):103216. doi: 10.1016/j.isci.2021.103216 (PMC8528732; doi:10.1016/j.isci.2021.103216)
Supplement: Document S1. Figures S1–S6 [file mmc1.pdf]

iScience, Volume *24*

# Supplemental information

## Modeling *Drosophila* gut microbe interactions reveals metabolic interconnectivity

**Jürgen W. Schönborn, Fiona A. Stewart, Kerstin Maas Enriquez, Irfan Akhtar, Andrea Droste, Silvio Waschina, and Mathias Beller**

## Supplemental figures and legends

A

| | Exp. growth rate [1/h] | | | In silico growth rate [1/h] | | |
|---|---|---|---|---|---|---|
| | MRS | ACE | HD | MRS | ACE | HD |
| ***L. plantarum* A2** | 0.26 | 0.11 | 0.13 | 0.24 | 0.26 | 0.36 |
| ***L. plantarum* B2** | 0.25 | 0.09 | 0.07 | 0.26 | 0.24 | 0.39 |
| ***L. brevis* B6** | 0.11 | 0.09 | 0.14 | 0.11 | 0.11 | 0.12 |
| ***A. indonesiensis* A4** | 0.10 | 0.07 | 0.20 | 0.29 | 0.30 | 0.08 |
| ***A. indonesiensis* A5** | 0.10 | 0.10 | 0.18 | 0.30 | 0.30 | 0.08 |
| ***A. pasteurianus* B5** | 0.05 | 0.05 | 0.20 | 0.28 | 0.30 | 0.09 |

B

$$\mu_{exp} = \frac{\ln(OD600_t) - \ln(OD600_{t_0})}{t - t_0}$$

$$\mu_{in\ silico} = \frac{\ln\left(\frac{biomass_t}{biomass_{t_0}}\right)}{t - t_0}$$

C

| | MRS | ACE | HD |
|---|---|---|---|
| ***L. plantarum* A2** | 0.02 | -0.15 | -0.24 |
| ***L. plantarum* B2** | -0.01 | -0.15 | -0.31 |
| ***L. brevis* B6** | 0.00 | -0.01 | 0.02 |
| ***A. indonesiensis* A4** | -0.19 | -0.23 | 0.12 |
| ***A. indonesiensis* A5** | -0.20 | -0.20 | 0.10 |
| ***A. pasteurianus* B5** | -0.23 | -0.25 | 0.11 |

*In silico* higher growth than experiment (delta ≥ - 0.2)

*In silico* elevated growth than experiment (delta > - 0.15 < - 0.2)

*In silico* and experimental growth comparable (delta ~ 0)

Experimental growth higher than *in silico* (delta ≥ 0.1)

**Figure S1. Growth estimate determination and comparison between wet-lab and *in silico* results, Related to Fig. 1 and Fig. 2.** (A) The growth rate of the six isolated bacteria was estimated on the three different media (MRS, ACE and HD) by wet-lab experiments (left part of the table) and using the computed biomass production amounts to calculate *in silico* derived growth rates (right side of the table). The formulas used are provided in (B). (C) We subtracted from the experimentally determined growth rate the *in silico* derived one to get a similarity measure (difference is “delta”). The color-coded delta values obtained are provided in the table.

| | | MRS | ACE | HD |
|---|---|---|---|---|
| **SUBSTRATE** | **CPD** | **Conc. [mM]** | **Conc. [mM]** | **Conc. [mM]** |
| GLUCOSE | cpd00027 | 111.00000 | 55.50000000 | - |
| SUCROSE | cpd00076 | - | - | 50 |
| GLUTAMINE | cpd00053 | 0.06842 | 0.10947657 | 12.47000000 |
| GLUTAMATE | cpd00023 | 19.47937 | 13.36912934 | 12.40000000 |
| PHENYLALANINE | cpd00066 | 4.64919 | 3.80168291 | 5.55000000 |
| HISTIDINE | cpd00119 | 2.27507 | 1.44367105 | 4.46000000 |
| LYSINE | cpd00039 | 7.98960 | 6.00588276 | 11.89000000 |
| METHIONINE | cpd00060 | 1.82976 | 1.13270777 | 2.27000000 |
| ARGININE | cpd00051 | 5.92423 | 6.18828932 | 8.17000000 |
| THREONINE | cpd00161 | 4.58361 | 2.45970450 | 10.86000000 |
| VALINE | cpd00156 | 8.02390 | 5.97524541 | 11.41000000 |
| TRYPTOPHAN | cpd00065 | 0.74426 | 0.41619742 | 2.15000000 |
| LEUCINE | cpd00107 | 9.41526 | 6.84607761 | 12.50000000 |
| ISOLEUCINE | cpd00322 | 5.72539 | 4.23115042 | 8.84000000 |
| ALANINE | cpd00035 | 17.15120 | 20.51857672 | 17.82000000 |
| ASPARTIC ACID | cpd00041 | 10.70624 | 8.82043576 | 6.32000000 |
| ASPARAGINE | cpd00132 | 0.68120 | 0.94610960 | 6.35000000 |
| GLYCINE | cpd00033 | 26.22885 | 34.96736379 | 14.43000000 |
| TYROSINE | cpd00069 | 1.58949 | 1.02654672 | 4.64000000 |
| CYSTEINE | cpd00084 | 0.18727 | 0.06658344 | 2.18000000 |
| SERINE | cpd00054 | 6.34694 | 3.35902560 | 7.23000000 |
| PROLINE | cpd00129 | 14.51403 | 12.85503344 | 4.90000000 |
| K+ | cpd00205 | 34.33960 | 7.49150219 | 22.00000 |
| CA2 | cpd00063 | 0.05115 | 0.03717750 | 1.14000 |
| ZN2 | cpd00034 | 0.01000 | 0.01000000 | 0.08700 |
| FE+2 | cpd10515 | 0.004895 | 0.00501 | 0.04495 |
| FE+3 | cpd10516 | 0.004895 | 0.00501 | 0.04495 |
| RIBOFLAVIN | cpd00220 | 0.01000 | 0.01000000 | 0.00186 |
| PHOSPHATE | cpd00009 | 6.34404 | 3.38629687 | 22.00000 |
| MAGNESIUM | cpd00254 | 1.90167 | 0.25735445 | 2.08000 |
| MN2+ | cpd00030 | 0.33128 | 0.01000000 | 0.00505 |
| BIOTINE | cpd00104 | 0.01000 | 0.01000000 | 0.00060 |
| CU2 | cpd00058 | 0.01000 | 0.01000000 | 0.01000 |
| CL- | cpd00099 | 3.28728 | 4.50289115 | 2.64830 |
| PANTOTHENATE | cpd00644 | 0.01000 | 0.01000000 | 0.04949 |
| THIAMIN | cpd00305 | 0.01000 | 0.01000000 | 0.00465 |
| SULFATE | cpd00048 | 1.77389 | 0.57464085 | 2.16687 |
| FOLIC ACID | cpd00393 | 0.01000 | 0.01000000 | 0.00113 |
| URIDINE | cpd00249 | 0.01000 | 0.01000000 | 0.24600 |
| INOSINE | cpd00246 | 0.01000 | 0.01000000 | 0.24200000 |
| NICOTINIC ACID | cpd00218 | 0.01000 | 0.01000000 | 0.06820000 |
| PYRIDOXINE | cpd30645 | 0.01000 | 0.01000000 | 0.01000000 |
| NA+ | cpd00971 | 4.68312 | 4.49582427 | 11.90500000 |
| O2 | cpd00007 | 0.10000 | 0.1 | 0.10000000 |
| WATER | cpd00001 | 100.00000 | 100.00000000 | 100.00000000 |
| MOLYBDATE | cpd11574 | 0.01000 | 0.01000000 | - |
| CO2+ | cpd00149 | 0.01000 | 0.01000000 | 0.01 |
| AMMONIUM | cpd00013 | 8.22335 | - | - |
| CITRATE | cpd00137 | 8.22335 | - | - |
| ACETATE | cpd00029 | 59.91674 | 52.40000000 | - |
| ETHANOL | cpd00363 | - | 85.60000000 | - |
| CHOLINE | cpd00098 | - | - | 0.35810 |
| MYO-INOSITOL | cpd00121 | - | - | 0.02800 |

**Figure S2. Parametrization of the media contents used in this study, Related to Table 1, STAR Methods, and Supplemental File 2.** The “CPD” ID refers to the compound descriptor used in the modeling simulations.

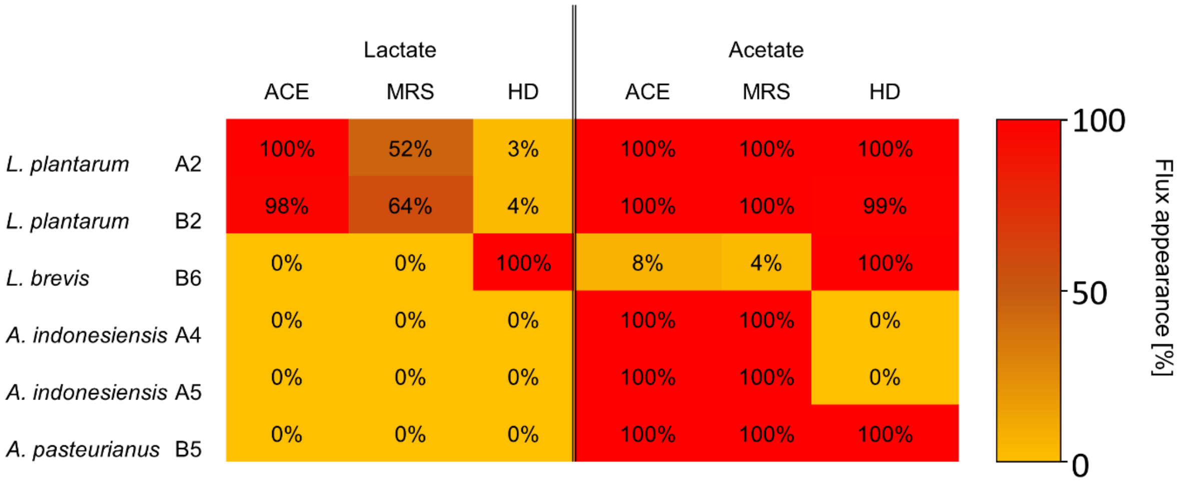


**Figure S3. Robustness analysis of the signature metabolite production of *Lactobacilli* and *Acetobacter* bacteria on the three different media, Related to Fig. 3.** Simulations were run 100 times and the production of lactate and acetate, respectively, was recorded. As a threshold, metabolite production had to surpass a flux rate of 0.1 nmol / h to count as “flux present”. The plots show the color-coded results with red color representing a high fraction of simulation runs with metabolite production and orange shades represent a low fraction of metabolite producing simulation runs.

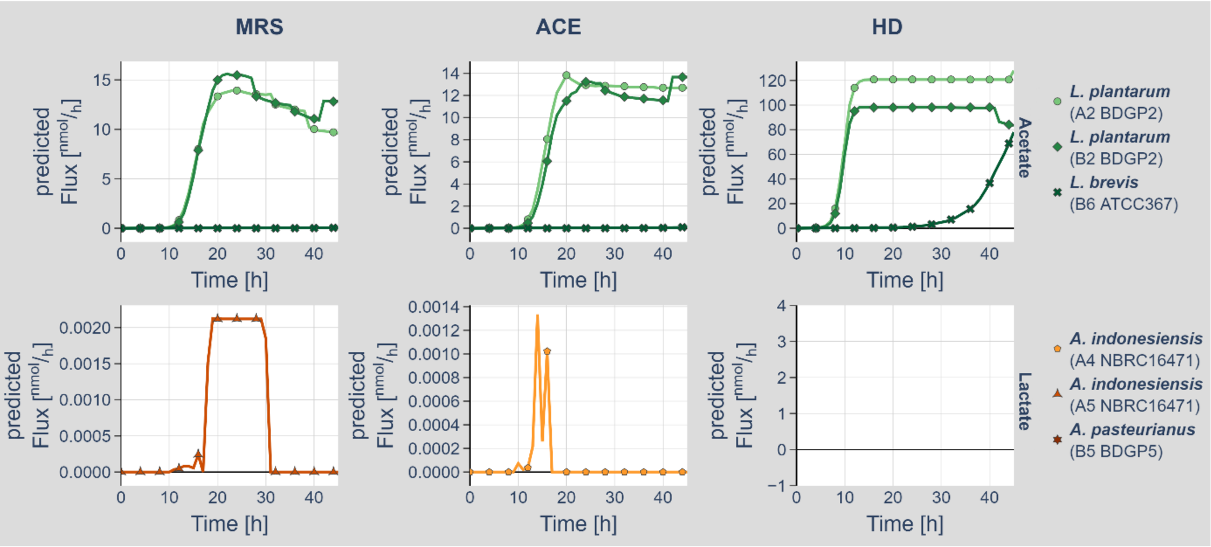


**Figure S4. Test for heterolactic behavior of *Acetobacter* and *Lactobacillus* models, Related to Fig. 3.** While all *Lactobacilli* were able to produce also acetate (upper row), the *Acetobacter* models failed to produce lactate exceeding our 0.1 nmol / h flux threshold.

| Name | Reac. ID | Single growth<br>Growth promoting for Aceto (Single metabolite add) | | |
|---|---|---|---|---|
| | | A4 | A5 | B5 |
| **Acetaldehyde** | EX_cpd00071_e0 | ++ | ++ | ++ |
| **ACTN (Acetoin)** | EX_cpd00361_e0 | ++ | ++ | ++ |
| **BDOH** | EX_cpd01947_e0 | ++ | ++ | ++ |
| **D-Alanine** | EX_cpd00117_e0 | ++ | ++ | // |
| **D-Fructose** | EX_cpd00082_e0 | ++ | ++ | ++ |
| **D-Ribose** | EX_cpd00105_e0 | ++ | ++ | ++ |
| **Fumarate** | EX_cpd00106_e0 | ++ | ++ | ++ |
| **H2O2** | EX_cpd00025_e0 | ++ | ++ | ++ |
| **Sorbitol** | EX_cpd00588_e0 | ++ | ++ | ++ |
| **Trehalose** | EX_cpd00794_e0 | ++ | ++ | ++ |
| (R)-1.2Propanediol | EX_cpd01861_e0 | // | // | // |
| Acetate | EX_cpd00029_e0 | // | // | // |
| AMP | EX_cpd00018_e0 | // | // | // |
| Arginine | EX_cpd00051_e0 | // | // | // |
| CMP | EX_cpd00046_e0 | // | // | // |
| CoA | EX_cpd00010_e0 | // | // | // |
| Cytosine | EX_cpd00307_e0 | // | // | // |
| Ethanol | EX_cpd00363_e0 | // | // | // |
| Fe2+ | EX_cpd10515_e0 | // | // | // |
| GABA | EX_cpd00281_e0 | // | // | // |
| GLCN Galactose | EX_cpd00222_e0 | // | // | // |
| GMP | EX_cpd00126_e0 | // | // | // |
| H2O | EX_cpd00001_e0 | // | // | // |
| H2S | EX_cpd00239_e0 | // | // | // |
| HEME | EX_cpd00028_e0 | // | // | // |
| Histidine | EX_cpd00119_e0 | // | // | // |
| Lactate | EX_cpd00159_e0 | // | // | // |
| Lysine | EX_cpd00039_e0 | // | // | // |
| Ornithine | EX_cpd00064_e0 | // | // | // |
| Pantothenate | EX_cpd00644_e0 | // | // | // |
| Phenylalanine | EX_cpd00066_e0 | // | // | // |
| Phosphate | EX_cpd00009_e0 | // | // | // |
| Proline | EX_cpd00129_e0 | // | // | // |
| Propionate | EX_cpd00141_e0 | // | // | // |
| Serine | EX_cpd00054_e0 | // | // | // |
| Succinate | EX_cpd00036_e0 | // | // | // |
| Tyrosine | EX_cpd00069_e0 | // | // | // |
| UMP | EX_cpd00091_e0 | // | // | // |
| Uracil | EX_cpd00092_e0 | // | // | // |
| Valine | EX_cpd00156_e0 | // | // | // |
| XAN | EX_cpd00309_e0 | // | // | // |

No growth rescue [Biomass < 700 pg]
Growth rescue [Biomass > 700 pg]

**Figure S5. Rescue of the *Acetobacter sp.* growth deficit in the HD by adding singular metabolites, Related to Fig. 5 and Fig. 6.** All metabolites which showed an exchange behavior in the combined growth simulations (cf. Figure 5A and supplemental file 2) were added individually to the HD simulations. The simulations were evaluated for a rescue of the growth deficit which is represented by red color in the table. The majority of added metabolites did not alter the biomass production of the model (represented by black color).

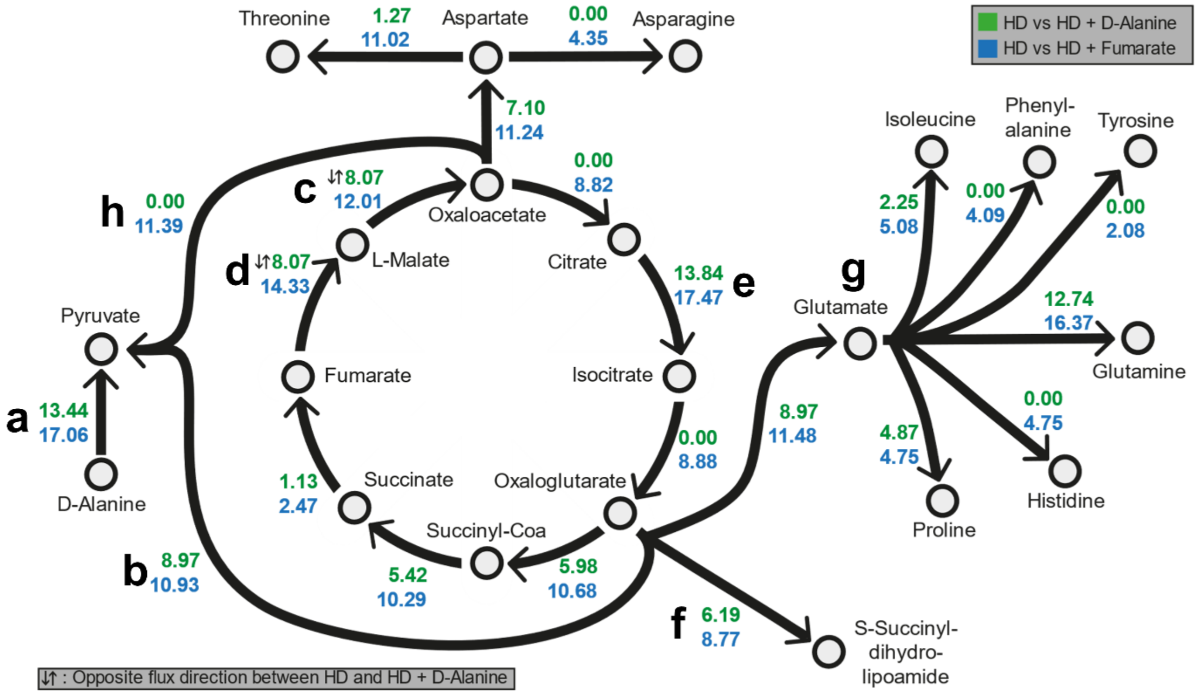


**Figure S6. Focused metabolic network with flux differences of *A. pasteurianus* (B5) growth modeled on HD or HD with D-Alanine or HD with fumarate, respectively, Related to Fig. 6.** Flux changes were calculated by subtracting the flux present on the HD from the value present in the HD with additive. The result was log normalized. Highlighted reactions are (if multiple reactions are provided, the individual flux values were combined to produce one summarizing value): a) L-Alanine racemase and L-alanine:glyoxylate aminotransferase, b) L-Alanine:2-oxoglutarate aminotransferase, c) Malate-dehydrogenase, d) Fumarase, e) Aconitase, f) 2-oxoglutarate dehydrogenase g) glutamate to isoleucine: L-Isoleucine:2-oxoglutarate aminotransferase; glutamate to phenylalanine: L-Phenylalanine:2-oxoglutarate aminotransferase; glutamate to tyrosine: L-tyrosine:2-oxoglutarate aminotransferase; glutamate to glutamine: L-Glutamate:ammonia ligase (ADP-forming) and L-glutamine:D-fructose-6-phosphate isomerase (deaminating); glutamate to histidine: L-Histidinol-phosphate phosphohydrolase and L-Histidinol:NAD+ oxidoreductase; glutamate to proline: ATP:L-glutamate 5-phosphotransferase and L-glutamate-5-

semialdehyde:NADP+ 5-oxidoreductase (phosphorylating) and L-glutamate 5-semialdehyde dehydratase and L-Proline:NADP+ 5-oxidoreductase, h) oxaloacetate carboxy-lyase (pyruvate-forming).
