# Supplementary material for: Modeling Drosophila gut microbe interactions reveals metabolic interconnectivity
Source: iScience. 2021 Oct 6;24(11):103216. doi: 10.1016/j.isci.2021.103216 (PMC8528732; doi:10.1016/j.isci.2021.103216)
Supplement: Data S3. MEMOTE analysis results for the quality control measurements of the genome-scale model quality. The zip folder contains all individual results as PDF files as well as interactive versions, which can be accessed via the html files, related to Table 1 [file mmc4.zip › MEMOTE/Interactive-HTML-Version/Acetobacter_indonesiensis_A4_memote.html]

MemoteReportApp

 Acetobacter\_indonesiensis\_A4  Expand AllReadme2021-04-15 12:44

Independent Section  Contains tests that are independent of the class of modeled organism, a model's complexity or types of identifiers that are used to describe its components. Parameterization or initialization of the network is not required. See readme for more details. 

## Consistency

Stoichiometric Consistency |

99.9% |

X3

Stoichiometric inconsistency violates universal constraints:
1. Molecular masses are always positive, and
2. On each side of a reaction the mass is conserved.
A single incorrectly defined reaction can lead to stoichiometric
inconsistency in the model, and consequently to unconserved metabolites.
Similar to insufficient constraints, this may give rise to cycles which
either produce mass from nothing or consume mass from the model.
Implementation:
This test first uses an implementation of the algorithm presented in
section 3.1 by Gevorgyan, A., M. G Poolman, and D. A Fell.
"Detection of Stoichiometric Inconsistencies in Biomolecular Models."
Bioinformatics 24, no. 19 (2008): 2245.
doi: 10.1093/bioinformatics/btn425 
Should the model be inconsistent, then the list of unconserved metabolites
is computed using the algorithm described in section 3.2 of the same
publication. In addition, the list of min unconservable sets is computed
using the algorithm described in section 3.3.

{"unconserved\_metabolites":["cpd00067\_e0","cpd00067\_c0"],"minimal\_unconservable\_sets":[["cpd00067\_c0"],["cpd00067\_e0"]]}

Mass Balance |

99.0% |

Charge Balance |

99.9% |

This will exclude biomass, exchange and demand reactions as they are
unbalanced by definition. It will also fail all reactions where at
least one metabolite does not have a charge defined.
In steady state, for each metabolite the sum of influx equals the sum
of efflux. Hence the net charges of both sides of any model reaction have
to be equal. Reactions where at least one metabolite does not have a
charge are not considered to be balanced, even though the remaining
metabolites participating in the reaction might be.
Implementation:
For each reaction that isn't a boundary or biomass reaction check if each
metabolite has a non-zero charge attribute and if so calculate if the
overall sum of charges of reactants and products is equal to zero.

A total of 2 (0.11%) reactions are charge unbalanced with at
least one of the metabolites not having a charge or the overall
charge not equal to 0: rxn14087\_c0, rxn06134\_c0

["rxn14087\_c0","rxn06134\_c0"]

Metabolite Connectivity |

100.0% |

Disconnected metabolites are not part of any reaction in the model. They
are most likely left-over from the reconstruction process, but may also
point to network and knowledge gaps.
Implementation:
Check for any metabolites of the cobra.Model object with emtpy reaction
attribute.

A total of 0 (0.00%) metabolites are not associated with any
reaction of the model:

[]

Unbounded Flux In Default Medium |

68.0% |

A large fraction of model reactions able to carry unlimited flux under
default conditions indicates problems with reaction directionality,
missing cofactors, incorrectly defined transport reactions and more.
Implementation:
Without changing the default constraints run flux variability analysis.
From the FVA results identify those reactions that carry flux equal to the
model's maximal or minimal flux.

A fraction of 32.01% of the non-blocked reactions (in total 218
reactions) can carry unbounded flux in the default model
condition. Unbounded reactions may be involved in
thermodynamically infeasible cycles: rxn00069\_c0, rxn00070\_c0,
rxn00083\_c0, rxn00086\_c0, rxn00191\_c0, ...

["rxn00069\_c0","rxn00070\_c0","rxn00083\_c0","rxn00086\_c0","rxn00191\_c0","rxn00225\_c0","rxn00283\_c0","rxn00289\_c0","rxn00324\_c0","rxn00336\_c0","rxn00453\_c0","rxn00512\_c0","rxn00536\_c0","rxn00543\_c0","rxn00555\_c0","rxn00558\_c0","rxn00559\_c0","rxn00604\_c0","rxn00605\_c0","rxn00656\_c0","rxn00657\_c0","rxn00669\_c0","rxn00677\_c0","rxn00684\_c0","rxn00686\_c0","rxn00704\_c0","rxn00763\_c0","rxn00765\_c0","rxn00777\_c0","rxn00778\_c0","rxn00781\_c0","rxn00785\_c0","rxn00797\_c0","rxn00856\_c0","rxn00927\_c0","rxn00952\_c0","rxn00957\_c0","rxn00958\_c0","rxn00973\_c0","rxn00974\_c0","rxn01011\_c0","rxn01013\_c0","rxn01056\_c0","rxn01138\_c0","rxn01171\_c0","rxn01174\_c0","rxn01200\_c0","rxn01226\_c0","rxn01290\_c0","rxn01291\_c0","rxn01299\_c0","rxn01358\_c0","rxn01387\_c0","rxn01388\_c0","rxn01474\_c0","rxn01475\_c0","rxn01541\_c0","rxn01545\_c0","rxn01548\_c0","rxn01601\_c0","rxn01602\_c0","rxn01615\_c0","rxn01618\_c0","rxn01649\_c0","rxn01683\_c0","rxn01684\_c0","rxn01816\_c0","rxn01831\_c0","rxn01835\_c0","rxn01840\_c0","rxn01841\_c0","rxn01867\_c0","rxn01868\_c0","rxn01879\_c0","rxn01937\_c0","rxn01943\_c0","rxn01944\_c0","rxn01975\_c0","rxn01977\_c0","rxn02185\_c0","rxn02186\_c0","rxn02209\_c0","rxn02226\_c0","rxn02227\_c0","rxn02260\_c0","rxn02261\_c0","rxn02287\_c0","rxn02302\_c0","rxn02364\_c0","rxn02365\_c0","rxn02380\_c0","rxn02382\_c0","rxn02527\_c0","rxn02528\_c0","rxn02565\_c0","rxn02566\_c0","rxn02789\_c0","rxn02811\_c0","rxn02925\_c0","rxn02926\_c0","rxn03060\_c0","rxn03061\_c0","rxn03068\_c0","rxn03335\_c0","rxn03336\_c0","rxn03340\_c0","rxn03341\_c0","rxn03343\_c0","rxn03344\_c0","rxn03435\_c0","rxn03436\_c0","rxn03798\_c0","rxn04443\_c0","rxn04676\_c0","rxn04678\_c0","rxn04937\_c0","rxn05005\_c0","rxn05006\_c0","rxn05207\_c0","rxn05210\_c0","rxn05215\_c0","rxn05221\_c0","rxn05297\_c0","rxn05298\_c0","rxn05312\_c0","rxn05488\_c0","rxn05494\_c0","rxn05496\_c0","rxn05582\_c0","rxn05595\_c0","rxn05605\_c0","rxn05638\_c0","rxn05651\_c0","rxn05957\_c0","rxn06252\_c0","rxn06253\_c0","rxn08062\_c0","rxn08386\_c0","rxn08387\_c0","rxn08390\_c0","rxn08391\_c0","rxn08392\_c0","rxn08393\_c0","rxn08394\_c0","rxn08395\_c0","rxn08398\_c0","rxn08399\_c0","rxn08582\_c0","rxn08661\_c0","rxn08764\_c0","rxn09402\_c0","rxn09519\_c0","rxn09882\_c0","rxn10770\_c0","rxn10820\_c0","rxn11551\_c0","rxn11552\_c0","rxn11810\_c0","rxn11811\_c0","rxn12191\_c0","rxn12193\_c0","rxn12204\_c0","rxn12303\_c0","rxn12649\_c0","rxn12811\_c0","rxn13660\_c0","rxn14077\_c0","rxn14120\_c0","rxn14246\_c0","rxn14359\_c0","rxn15045\_c0","rxn15046\_c0","rxn15064\_c0","rxn15072\_c0","rxn15114\_c0","rxn15115\_c0","rxn15118\_c0","rxn15122\_c0","rxn15123\_c0","rxn15126\_c0","rxn15127\_c0","rxn15162\_c0","rxn15171\_c0","rxn15172\_c0","rxn15228\_c0","rxn15229\_c0","rxn15238\_c0","rxn15239\_c0","rxn15240\_c0","rxn15241\_c0","rxn15248\_c0","rxn15270\_c0","rxn15271\_c0","rxn15278\_c0","rxn15279\_c0","rxn15280\_c0","rxn15281\_c0","rxn15306\_c0","rxn15307\_c0","rxn15317\_c0","rxn15319\_c0","rxn15341\_c0","rxn15345\_c0","rxn15346\_c0","rxn15362\_c0","rxn15364\_c0","rxn15399\_c0","rxn15410\_c0","rxn15491\_c0","rxn15511\_c0","rxn15630\_c0","rxn15640\_c0","rxn15660\_c0","rxn16149\_c0","rxn38971\_c0","rxn38972\_c0","rxn00193\_c0","rxn09120\_c0"]
 ["rxn00069\_c0","rxn00070\_c0","rxn00083\_c0","rxn00086\_c0","rxn00191\_c0","rxn00225\_c0","rxn00283\_c0","rxn00289\_c0","rxn00324\_c0","rxn00336\_c0","rxn00453\_c0","rxn00512\_c0","rxn00536\_c0","rxn00543\_c0","rxn00555\_c0","rxn00558\_c0","rxn00559\_c0","rxn00604\_c0","rxn00605\_c0","rxn00656\_c0","rxn00657\_c0","rxn00669\_c0","rxn00677\_c0","rxn00684\_c0","rxn00686\_c0","rxn00704\_c0","rxn00763\_c0","rxn00765\_c0","rxn00777\_c0","rxn00778\_c0","rxn00781\_c0","rxn00785\_c0","rxn00797\_c0","rxn00856\_c0","rxn00927\_c0","rxn00952\_c0","rxn00957\_c0","rxn00958\_c0","rxn00973\_c0","rxn00974\_c0","rxn01011\_c0","rxn01013\_c0","rxn01056\_c0","rxn01138\_c0","rxn01171\_c0","rxn01174\_c0","rxn01200\_c0","rxn01226\_c0","rxn01290\_c0","rxn01291\_c0","rxn01299\_c0","rxn01358\_c0","rxn01387\_c0","rxn01388\_c0","rxn01474\_c0","rxn01475\_c0","rxn01541\_c0","rxn01545\_c0","rxn01548\_c0","rxn01601\_c0","rxn01602\_c0","rxn01615\_c0","rxn01618\_c0","rxn01649\_c0","rxn01683\_c0","rxn01684\_c0","rxn01816\_c0","rxn01831\_c0","rxn01835\_c0","rxn01840\_c0","rxn01841\_c0","rxn01867\_c0","rxn01868\_c0","rxn01879\_c0","rxn01937\_c0","rxn01943\_c0","rxn01944\_c0","rxn01975\_c0","rxn01977\_c0","rxn02185\_c0","rxn02186\_c0","rxn02209\_c0","rxn02226\_c0","rxn02227\_c0","rxn02260\_c0","rxn02261\_c0","rxn02287\_c0","rxn02302\_c0","rxn02364\_c0","rxn02365\_c0","rxn02380\_c0","rxn02382\_c0","rxn02527\_c0","rxn02528\_c0","rxn02565\_c0","rxn02566\_c0","rxn02789\_c0","rxn02811\_c0","rxn02925\_c0","rxn02926\_c0","rxn03060\_c0","rxn03061\_c0","rxn03068\_c0","rxn03335\_c0","rxn03336\_c0","rxn03340\_c0","rxn03341\_c0","rxn03343\_c0","rxn03344\_c0","rxn03435\_c0","rxn03436\_c0","rxn03798\_c0","rxn04443\_c0","rxn04676\_c0","rxn04678\_c0","rxn04937\_c0","rxn05005\_c0","rxn05006\_c0","rxn05207\_c0","rxn05210\_c0","rxn05215\_c0","rxn05221\_c0","rxn05297\_c0","rxn05298\_c0","rxn05312\_c0","rxn05488\_c0","rxn05494\_c0","rxn05496\_c0","rxn05582\_c0","rxn05595\_c0","rxn05605\_c0","rxn05638\_c0","rxn05651\_c0","rxn05957\_c0","rxn06252\_c0","rxn06253\_c0","rxn08062\_c0","rxn08386\_c0","rxn08387\_c0","rxn08390\_c0","rxn08391\_c0","rxn08392\_c0","rxn08393\_c0","rxn08394\_c0","rxn08395\_c0","rxn08398\_c0","rxn08399\_c0","rxn08582\_c0","rxn08661\_c0","rxn08764\_c0","rxn09402\_c0","rxn09519\_c0","rxn09882\_c0","rxn10770\_c0","rxn10820\_c0","rxn11551\_c0","rxn11552\_c0","rxn11810\_c0","rxn11811\_c0","rxn12191\_c0","rxn12193\_c0","rxn12204\_c0","rxn12303\_c0","rxn12649\_c0","rxn12811\_c0","rxn13660\_c0","rxn14077\_c0","rxn14120\_c0","rxn14246\_c0","rxn14359\_c0","rxn15045\_c0","rxn15046\_c0","rxn15064\_c0","rxn15072\_c0","rxn15114\_c0","rxn15115\_c0","rxn15118\_c0","rxn15122\_c0","rxn15123\_c0","rxn15126\_c0","rxn15127\_c0","rxn15162\_c0","rxn15171\_c0","rxn15172\_c0","rxn15228\_c0","rxn15229\_c0","rxn15238\_c0","rxn15239\_c0","rxn15240\_c0","rxn15241\_c0","rxn15248\_c0","rxn15270\_c0","rxn15271\_c0","rxn15278\_c0","rxn15279\_c0","rxn15280\_c0","rxn15281\_c0","rxn15306\_c0","rxn15307\_c0","rxn15317\_c0","rxn15319\_c0","rxn15341\_c0","rxn15345\_c0","rxn15346\_c0","rxn15362\_c0","rxn15364\_c0","rxn15399\_c0","rxn15410\_c0","rxn15491\_c0","rxn15511\_c0","rxn15630\_c0","rxn15640\_c0","rxn15660\_c0","rxn16149\_c0","rxn38971\_c0","rxn38972\_c0","rxn00193\_c0","rxn09120\_c0"]

---

Sub Total |

95% |

X3

The Sub Total is the result of the following calculation. For more information please click on "Readme" in the top left of the report.

99.89+99.05+99.89+67.99+(1⋅100)+(5⋅100)=95.2399.89+99.05+99.89+67.99+(1⋅100)+(5⋅100)=95.23

## Annotation - Metabolites

Presence of Metabolite Annotation |

100.0% |

Metabolite Annotations Per Database | Info |

Specific database cross-references are paramount to mapping information.
To provide references to as many databases as possible helps to make the
metabolic model more accessible to other researchers. This does not only
facilitate the use of a model in a broad array of computational pipelines,
it also promotes the metabolic model itself to become an organism-specific
knowledge base.
For this test to pass, each metabolite annotation should contain
cross-references to a number of databases. The currently selection is
listed in `annotation.py`, but an ongoing discussion can be found at
https://github.com/opencobra/memote/issues/332. For each database this
test checks for the presence of its corresponding namespace ID to comply
with the MIRIAM guidelines i.e. they have to match those defined on
https://identifiers.org/.
Since each database is quite different and some potentially incomplete, it
may not be feasible to achieve 100% coverage for each of them. Generally
it should be possible, however, to obtain cross-references to at least
one of the databases for all metabolites consistently.
Implementation:
Check if the keys of the annotation attribute of each cobra.Metabolite of
the model match with a selection of common biochemical databases. The
annotation attribute of cobrapy components is a dictionary of
key:value pairs.

 pubchem.compound |

0.0% |

 kegg.compound |

86.1% |

 seed.compound |

100.0% |

 inchikey |

81.2% |

 inchi |

0.0% |

 chebi |

87.0% |

 hmdb |

59.4% |

 reactome |

34.0% |

 metanetx.chemical |

99.9% |

 bigg.metabolite |

66.6% |

 biocyc |

77.3% |

Metabolite Annotation Conformity Per Database | Info |

 pubchem.compound |

0.0% |

 kegg.compound |

100.0% |

 seed.compound |

100.0% |

 inchikey |

100.0% |

 inchi |

0.0% |

 chebi |

100.0% |

 hmdb |

100.0% |

 reactome |

100.0% |

 metanetx.chemical |

99.9% |

 bigg.metabolite |

100.0% |

 biocyc |

100.0% |

Uniform Metabolite Identifier Namespace |

100.0% |

---

Sub Total |

86% |

The Sub Total is the result of the following calculation. For more information please click on "Readme" in the top left of the report.

86.10+81.23+87.01+59.44+33.98+99.94+66.65+77.31+99.94+(11⋅100)+(4⋅0)(24⋅100)=86.1786.10+81.23+87.01+59.44+33.98+99.94+66.65+77.31+99.94+(11⋅100)+(4⋅0)(24⋅100)=86.17

## Annotation - Reactions

Presence of Reaction Annotation |

100.0% |

This test checks if any annotations at all are present in the SBML
annotations field for each reaction, irrespective of the type of
annotation i.e. specific database cross-references, ontology terms,
additional information. For this test to pass the model is expected to
have reactions and each of them should have some form of annotation.
Implementation:
Check if the annotation attribute of each cobra.Reaction object of the
model is unset or empty.

A total of 0 reactions (0.00%) lack any form of annotation:

[]

Reaction Annotations Per Database | Info |

 rhea |

0.0% |

 kegg.reaction |

53.5% |

 seed.reaction |

92.0% |

|The following 154 reactions (7.98%) lack annotation for seed.reaction:
rxn90001\_c0, rxn90017\_c0, rxn90020\_c0, rxn90051\_c0, rxn90022\_c0, ...
|  |

["rxn90001\_c0","rxn90017\_c0","rxn90020\_c0","rxn90051\_c0","rxn90022\_c0","rxn90023\_c0","EX\_cpd00009\_e0","EX\_cpd00023\_e0","EX\_cpd00028\_e0","EX\_cpd00030\_e0","EX\_cpd00034\_e0","EX\_cpd00041\_e0","EX\_cpd00048\_e0","EX\_cpd00053\_e0","EX\_cpd00118\_e0","EX\_cpd00119\_e0","EX\_cpd00149\_e0","EX\_cpd00209\_e0","EX\_cpd00268\_e0","EX\_cpd00637\_e0","EX\_cpd10516\_e0","EX\_cpd00067\_e0","EX\_cpd00309\_e0","EX\_cpd00130\_e0","EX\_cpd00971\_e0","EX\_cpd00137\_e0","EX\_cpd00035\_e0","EX\_cpd00060\_e0","EX\_cpd00129\_e0","EX\_cpd00084\_e0","EX\_cpd00039\_e0","EX\_cpd00066\_e0","EX\_cpd00013\_e0","EX\_cpd00126\_e0","EX\_cpd00122\_e0","EX\_cpd00029\_e0","EX\_cpd00117\_e0","EX\_cpd00018\_e0","EX\_cpd00224\_e0","EX\_cpd00132\_e0","EX\_cpd01012\_e0","EX\_cpd00058\_e0","EX\_cpd10515\_e0","EX\_cpd00106\_e0","EX\_cpd00281\_e0","EX\_cpd00108\_e0","EX\_cpd00222\_e0","EX\_cpd00027\_e0","EX\_cpd00540\_e0","EX\_cpd00033\_e0","EX\_cpd00205\_e0","EX\_cpd00159\_e0","EX\_cpd01132\_e0","EX\_cpd00588\_e0","EX\_cpd00550\_e0","EX\_cpd00036\_e0","EX\_cpd00154\_e0","EX\_cpd00211\_e0","EX\_cpd00366\_e0","EX\_cpd12777\_e0","EX\_cpd00001\_e0","EX\_cpd01107\_e0","EX\_cpd01741\_e0","EX\_cpd03847\_e0","EX\_cpd15298\_e0","EX\_cpd00214\_e0","EX\_cpd15237\_e0","EX\_cpd01080\_e0","EX\_cpd15269\_e0","EX\_cpd01113\_e0","EX\_cpd03846\_e0","EX\_cpd00531\_e0","EX\_cpd00573\_e0","EX\_cpd00244\_e0","EX\_cpd04097\_e0","EX\_cpd00185\_e0","EX\_cpd00006\_e0","EX\_cpd00003\_e0","EX\_cpd11451\_e0","EX\_cpd11606\_e0","EX\_cpd17026\_e0","EX\_cpd17027\_e0","EX\_cpd00557\_e0","EX\_cpd00011\_e0","EX\_cpd00007\_e0","EX\_cpd01981\_e0","EX\_cpd00141\_e0","EX\_cpd00073\_e0","EX\_cpd01861\_e0","EX\_cpd00453\_e0","EX\_cpd00071\_e0","EX\_cpd00150\_e0","EX\_cpd08021\_e0","EX\_cpd00450\_e0","EX\_cpd00363\_e0","EX\_cpd00055\_e0","EX\_cpd00448\_e0","EX\_cpd00025\_e0","EX\_cpd00239\_e0","EX\_cpd11640\_e0","EX\_cpd00359\_e0","EX\_cpd11574\_e0","EX\_cpd00659\_e0","EX\_cpd00418\_e0","EX\_cpd00371\_e0","EX\_cpd00811\_e0","EX\_cpd01024\_e0","EX\_cpd00204\_e0","EX\_cpd00528\_e0","EX\_cpd00361\_e0","EX\_cpd01947\_e0","EX\_cpd11632\_e0","EX\_cpd03662\_e0","EX\_cpd00178\_e0","EX\_cpd01618\_e0","bio1","DM\_cpd01042\_c0","EX\_cpd00051\_e0","EX\_cpd00161\_e0","EX\_cpd00156\_e0","EX\_cpd00065\_e0","EX\_cpd00107\_e0","EX\_cpd00322\_e0","EX\_cpd00069\_e0","EX\_cpd00054\_e0","EX\_cpd00063\_e0","EX\_cpd00220\_e0","EX\_cpd00393\_e0","EX\_cpd00254\_e0","EX\_cpd00104\_e0","EX\_cpd00099\_e0","EX\_cpd00644\_e0","EX\_cpd00305\_e0","EX\_cpd00249\_e0","EX\_cpd00246\_e0","EX\_cpd00218\_e0","EX\_cpd30645\_e0","EX\_cpd11416\_c0","rxn90019\_c0","EX\_cpd01048\_e0","EX\_cpd11595\_e0","EX\_cpd00082\_e0","EX\_cpd00121\_e0","EX\_cpd00794\_e0","EX\_cpd00047\_e0","EX\_cpd00064\_e0","EX\_cpd00100\_e0","EX\_cpd00105\_e0","EX\_cpd00010\_e0","EX\_cpd00080\_e0","EX\_cpd00092\_e0","EX\_cpd00307\_e0","EX\_cpd00046\_e0","EX\_cpd00091\_e0"]

 metanetx.reaction |

62.1% |

 bigg.reaction |

39.4% |

 reactome |

0.0% |

 ec-code |

80.8% |

 brenda |

0.0% |

 biocyc |

42.1% |

Reaction Annotation Conformity Per Database | Info |

 rhea |

0.0% |

 kegg.reaction |

100.0% |

 seed.reaction |

100.0% |

|A total of 0 reaction annotations (0.00%) do not match the
regular expression patterns defined on identifiers.org for the
seed.reaction database:
|  |

[]

 metanetx.reaction |

100.0% |

 bigg.reaction |

100.0% |

 reactome |

0.0% |

 ec-code |

99.6% |

 brenda |

0.0% |

 biocyc |

100.0% |

Uniform Reaction Identifier Namespace |

100.0% |

---

Sub Total |

77% |

The Sub Total is the result of the following calculation. For more information please click on "Readme" in the top left of the report.

53.55+92.02+62.09+39.41+80.84+42.05+99.59+(7⋅100)+(6⋅0)(20⋅100)=76.9353.55+92.02+62.09+39.41+80.84+42.05+99.59+(7⋅100)+(6⋅0)(20⋅100)=76.93

## Annotation - Genes

Presence of Gene Annotation |

0.0% |

Gene Annotations Per Database | Info |

 refseq |

0.0% |

 uniprot |

0.0% |

 ecogene |

0.0% |

 kegg.genes |

0.0% |

 ncbigi |

0.0% |

 ncbigene |

0.0% |

 ncbiprotein |

0.0% |

 ccds |

0.0% |

 hprd |

0.0% |

 asap |

0.0% |

Gene Annotation Conformity Per Database | Info |

 refseq |

0.0% |

 uniprot |

0.0% |

 ecogene |

0.0% |

 kegg.genes |

0.0% |

 ncbigi |

0.0% |

 ncbigene |

0.0% |

 ncbiprotein |

0.0% |

 ccds |

0.0% |

 hprd |

0.0% |

 asap |

0.0% |

---

Sub Total |

0% |

The Sub Total is the result of the following calculation. For more information please click on "Readme" in the top left of the report.

(21⋅0)(21⋅100)=0.00(21⋅0)(21⋅100)=0.00

## Annotation - SBO Terms

Metabolite General SBO Presence |

100.0% |

Metabolite SBO:0000247 Presence |

100.0% |

Reaction General SBO Presence |

100.0% |

Metabolic Reaction SBO:0000176 Presence |

99.9% |

Transport Reaction SBO:0000185 Presence |

63.7% |

Exchange Reaction SBO:0000627 Presence |

100.0% |

Demand Reaction SBO:0000628 Presence |

100.0% |

Sink Reactions SBO:0000632 Presence |

Skipped |

Gene General SBO Presence |

0.0% |

Gene SBO:0000243 Presence |

0.0% |

Biomass Reactions SBO:0000629 Presence |

100.0% |

---

Sub Total |

69% |

X2

The Sub Total is the result of the following calculation. For more information please click on "Readme" in the top left of the report.

99.87+63.73+(6⋅100)+(3⋅0)(11⋅100)=69.4299.87+63.73+(6⋅100)+(3⋅0)(11⋅100)=69.42

---

---

Total Score |

77% |

The Total Score is the result of the following calculation. For more information please click on "Readme" in the top left of the report.

(3⋅95.23)+(1⋅86.17)+(1⋅76.93)+(1⋅0.00)+(2⋅69.42)(3⋅100)+(1⋅100)+(1⋅100)+(1⋅100)+(2⋅100)=77.40(3⋅95.23)+(1⋅86.17)+(1⋅76.93)+(1⋅0.00)+(2⋅69.42)(3⋅100)+(1⋅100)+(1⋅100)+(1⋅100)+(2⋅100)=77.40

---

Total Score 

77%

Score per Category 

Export

0%10%20%30%40%50%60%70%80%90%100%scoreconsistencyannotation\_metannotation\_rxnannotation\_geneannotation\_sbosection

Specific Section  Covers general statistics and specific aspects of a metabolic network that are not universally applicable. See readme for more details. 

SBML

SBML Level and Version |

Errored |

This test reports if the model file is represented in the latest edition
(level) of the Systems Biology Markup Language (SBML) which is Level 3,
and at least version 1.
Implementation:
The level and version are parsed directly from the SBML document.

null

FBC enabled |

Errored |

The Flux Balance Constraints (FBC) Package extends SBML with structured
and semantic descriptions for domain-specific model components such as
flux bounds, multiple linear objective functions, gene-protein-reaction
associations, metabolite chemical formulas, charge and related annotations
which are relevant for parameterized GEMs and FBA models. The SBML and
constraint-based modeling communities collaboratively develop this package
and update it based on user input.
Implementation:
Parse the state of the FBC plugin from the SBML document.

null

Basic Information

Model Identifier |

Acetobacter\_indonesiensis\_A4 |

Total Metabolites |

1,763 |

Total Reactions |

1,931 |

Total Genes |

631 |

Total Compartments |

3 |

Metabolic Coverage |

3.06 |

Metabolite Information

Unique Metabolites |

1,618 |

Duplicate Metabolites in Identical Compartments |

18 |

Metabolites without Charge |

0 |

Metabolites without Formula |

0 |

Medium Components |

50 |

Reaction Information

Purely Metabolic Reactions |

1,588 |

Purely Metabolic Reactions with Constraints |

120 |

Transport Reactions |

193 |

Transport Reactions with Constraints |

3 |

Thermodynamic Reversibility of Purely Metabolic Reactions |

0.40 |

Reactions With Partially Identical Annotations |

0.01 |

Duplicate Reactions |

0.01 |

Reactions With Identical Genes |

0.60 |

Gene-Protein-Reaction (GPR) Associations

Reactions without GPR |

329 |

Fraction of Transport Reactions without GPR |

0.40 |

Enzyme Complexes |

78 |

Biomass

Biomass Reactions Identified |

1 |

Biomass Consistency |

Errored |

Biomass Production In Default Medium |

0.56 |

Unrealistic Growth Rate In Default Medium |

false |

Biomass Production In Complete Medium |

109.42 |

Blocked Biomass Precursors In Default Medium |

3 |

Blocked Biomass Precursors In Complete Medium |

3 |

Ratio of Direct Metabolites in Biomass Reaction |

0.12 |

Number of Missing Essential Biomass Precursors |

3 |

Energy Metabolism

Non-Growth Associated Maintenance Reaction |

1 |

Growth-associated Maintenance in Biomass Reaction |

true |

Number of Reversible Oxygen-Containing Reactions |

6 |

Erroneous Energy-generating Cycles | Info |

 MNXM3 |

Skipped |

 MNXM63 |

Skipped |

 MNXM51 |

Skipped |

 MNXM121 |

Skipped |

 MNXM423 |

Skipped |

 MNXM6 |

Skipped |

 MNXM10 |

Skipped |

 MNXM38 |

Skipped |

 MNXM208 |

Skipped |

 MNXM191 |

Skipped |

 MNXM223 |

Skipped |

 MNXM7517 |

Skipped |

 MNXM12233 |

Skipped |

 MNXM558 |

Skipped |

 MNXM21 |

Skipped |

 MNXM89557 |

Skipped |

Network Topology

Universally Blocked Reactions |

837 |

Orphan Metabolites |

209 |

Dead-end Metabolites |

207 |

Stoichiometrically Balanced Cycles |

265 |

Metabolite Production In Complete Medium |

922 |

Metabolite Consumption In Complete Medium |

959 |

Matrix Conditioning

Ratio Min/Max Non-Zero Coefficients |

0.00 |

Independent Conservation Relations |

321 |

Rank |

1442 |

Degrees Of Freedom |

489 |

Experimental Data Comparison

Growth Prediction |

Skipped |

Gene Essentiality Prediction |

Skipped |

Misc. Tests

Environment Python Version 3.6.12 Platform Linux Memote Version 0.11.1

Package Versions

{"click-log":"0.3.2","requests":"2.24.0","click":"7.1.2","travis-encrypt":"1.1.2","click-configfile":"0.2.3","importlib-resources":"3.0.0","pytest":"6.0.1","ruamel.yaml":"0.16.10","cobra":"0.18.1","goodtables":"2.5.0","equilibrator-api":"0.1.26","Jinja2":"2.11.2","six":"1.15.0","cookiecutter":"1.7.2","sqlalchemy":"1.3.19","future":"0.18.2","lxml":"4.5.2","gitpython":"3.1.7","depinfo":"1.5.4","numpydoc":"1.1.0","pylru":"1.2.0","pandas":"1.1.0","sympy":"1.6.2","memote":"0.11.1","pip":"20.2.2","setuptools":"49.6.0","wheel":"0.35.1"}
