# Supplementary material for: Modeling Drosophila gut microbe interactions reveals metabolic interconnectivity
Source: iScience. 2021 Oct 6;24(11):103216. doi: 10.1016/j.isci.2021.103216 (PMC8528732; doi:10.1016/j.isci.2021.103216)
Supplement: Data S3. MEMOTE analysis results for the quality control measurements of the genome-scale model quality. The zip folder contains all individual results as PDF files as well as interactive versions, which can be accessed via the html files, related to Table 1 [file mmc4.zip › MEMOTE/Interactive-HTML-Version/Acetobacter_pasteurianus_B5_memote.html]

MemoteReportApp

 Acetobacter\_pasteurianus\_B5  Expand AllReadme2021-04-16 08:10

Independent Section  Contains tests that are independent of the class of modeled organism, a model's complexity or types of identifiers that are used to describe its components. Parameterization or initialization of the network is not required. See readme for more details. 

## Consistency

Stoichiometric Consistency |

99.9% |

X3

Mass Balance |

99.0% |

Charge Balance |

99.9% |

Metabolite Connectivity |

100.0% |

Unbounded Flux In Default Medium |

71.1% |

---

Sub Total |

96% |

X3

The Sub Total is the result of the following calculation. For more information please click on "Readme" in the top left of the report.

99.88+99.03+99.94+71.10+(1⋅100)+(5⋅100)=95.6799.88+99.03+99.94+71.10+(1⋅100)+(5⋅100)=95.67

## Annotation - Metabolites

Presence of Metabolite Annotation |

100.0% |

Metabolite Annotations Per Database | Info |

 pubchem.compound |

0.0% |

 kegg.compound |

86.0% |

 seed.compound |

100.0% |

 inchikey |

81.1% |

 inchi |

0.0% |

 chebi |

86.8% |

 hmdb |

58.6% |

 reactome |

33.1% |

 metanetx.chemical |

99.9% |

 bigg.metabolite |

66.5% |

 biocyc |

77.9% |

Metabolite Annotation Conformity Per Database | Info |

 pubchem.compound |

0.0% |

 kegg.compound |

100.0% |

 seed.compound |

100.0% |

 inchikey |

100.0% |

 inchi |

0.0% |

 chebi |

100.0% |

 hmdb |

100.0% |

 reactome |

100.0% |

 metanetx.chemical |

99.9% |

 bigg.metabolite |

100.0% |

 biocyc |

100.0% |

Uniform Metabolite Identifier Namespace |

100.0% |

---

Sub Total |

86% |

The Sub Total is the result of the following calculation. For more information please click on "Readme" in the top left of the report.

86.01+81.11+86.85+58.58+33.05+99.94+66.53+77.94+99.94+(11⋅100)+(4⋅0)(24⋅100)=86.1486.01+81.11+86.85+58.58+33.05+99.94+66.53+77.94+99.94+(11⋅100)+(4⋅0)(24⋅100)=86.14

## Annotation - Reactions

Presence of Reaction Annotation |

100.0% |

Reaction Annotations Per Database | Info |

 rhea |

0.0% |

 kegg.reaction |

53.7% |

 seed.reaction |

91.9% |

 metanetx.reaction |

61.8% |

 bigg.reaction |

39.0% |

 reactome |

0.0% |

 ec-code |

80.3% |

 brenda |

0.0% |

 biocyc |

41.8% |

Reaction Annotation Conformity Per Database | Info |

 rhea |

0.0% |

 kegg.reaction |

100.0% |

 seed.reaction |

100.0% |

 metanetx.reaction |

100.0% |

 bigg.reaction |

100.0% |

 reactome |

0.0% |

 ec-code |

99.7% |

 brenda |

0.0% |

 biocyc |

100.0% |

Uniform Reaction Identifier Namespace |

100.0% |

---

Sub Total |

77% |

The Sub Total is the result of the following calculation. For more information please click on "Readme" in the top left of the report.

53.73+91.87+61.80+39.03+80.35+41.82+99.67+(7⋅100)+(6⋅0)(20⋅100)=76.9053.73+91.87+61.80+39.03+80.35+41.82+99.67+(7⋅100)+(6⋅0)(20⋅100)=76.90

## Annotation - Genes

Presence of Gene Annotation |

0.0% |

Gene Annotations Per Database | Info |

 refseq |

0.0% |

 uniprot |

0.0% |

 ecogene |

0.0% |

 kegg.genes |

0.0% |

 ncbigi |

0.0% |

 ncbigene |

0.0% |

 ncbiprotein |

0.0% |

 ccds |

0.0% |

 hprd |

0.0% |

 asap |

0.0% |

Gene Annotation Conformity Per Database | Info |

 refseq |

0.0% |

 uniprot |

0.0% |

 ecogene |

0.0% |

 kegg.genes |

0.0% |

 ncbigi |

0.0% |

 ncbigene |

0.0% |

 ncbiprotein |

0.0% |

 ccds |

0.0% |

 hprd |

0.0% |

 asap |

0.0% |

---

Sub Total |

0% |

The Sub Total is the result of the following calculation. For more information please click on "Readme" in the top left of the report.

(21⋅0)(21⋅100)=0.00(21⋅0)(21⋅100)=0.00

## Annotation - SBO Terms

Metabolite General SBO Presence |

100.0% |

Metabolite SBO:0000247 Presence |

100.0% |

Reaction General SBO Presence |

100.0% |

Metabolic Reaction SBO:0000176 Presence |

99.9% |

Transport Reaction SBO:0000185 Presence |

62.8% |

Exchange Reaction SBO:0000627 Presence |

100.0% |

Demand Reaction SBO:0000628 Presence |

100.0% |

Sink Reactions SBO:0000632 Presence |

Skipped |

Gene General SBO Presence |

0.0% |

Gene SBO:0000243 Presence |

0.0% |

Biomass Reactions SBO:0000629 Presence |

100.0% |

---

Sub Total |

69% |

X2

The Sub Total is the result of the following calculation. For more information please click on "Readme" in the top left of the report.

99.86+62.84+(6⋅100)+(3⋅0)(11⋅100)=69.3499.86+62.84+(6⋅100)+(3⋅0)(11⋅100)=69.34

---

---

Total Score |

78% |

The Total Score is the result of the following calculation. For more information please click on "Readme" in the top left of the report.

(3⋅95.67)+(1⋅86.14)+(1⋅76.90)+(1⋅0.00)+(2⋅69.34)(3⋅100)+(1⋅100)+(1⋅100)+(1⋅100)+(2⋅100)=77.53(3⋅95.67)+(1⋅86.14)+(1⋅76.90)+(1⋅0.00)+(2⋅69.34)(3⋅100)+(1⋅100)+(1⋅100)+(1⋅100)+(2⋅100)=77.53

---

Total Score 

78%

Score per Category 

Export

0%10%20%30%40%50%60%70%80%90%100%scoreconsistencyannotation\_metannotation\_rxnannotation\_geneannotation\_sbosection

Specific Section  Covers general statistics and specific aspects of a metabolic network that are not universally applicable. See readme for more details. 

SBML

SBML Level and Version |

Errored |

FBC enabled |

Errored |

Basic Information

Model Identifier |

Acetobacter\_pasteurianus\_B5 |

Total Metabolites |

1,673 |

Total Reactions |

1,796 |

Total Genes |

580 |

Total Compartments |

3 |

Metabolic Coverage |

3.10 |

Metabolite Information

Unique Metabolites |

1,536 |

Duplicate Metabolites in Identical Compartments |

18 |

Metabolites without Charge |

0 |

Metabolites without Formula |

0 |

Medium Components |

50 |

Reaction Information

Purely Metabolic Reactions |

1,468 |

Purely Metabolic Reactions with Constraints |

112 |

Transport Reactions |

183 |

Transport Reactions with Constraints |

3 |

Thermodynamic Reversibility of Purely Metabolic Reactions |

0.40 |

Reactions With Partially Identical Annotations |

0.01 |

Duplicate Reactions |

0.01 |

Reactions With Identical Genes |

0.59 |

Gene-Protein-Reaction (GPR) Associations

Reactions without GPR |

319 |

Fraction of Transport Reactions without GPR |

0.43 |

Enzyme Complexes |

74 |

Biomass

Biomass Reactions Identified |

1 |

Biomass Consistency |

Errored |

Biomass Production In Default Medium |

0.41 |

Unrealistic Growth Rate In Default Medium |

false |

Biomass Production In Complete Medium |

65.13 |

Blocked Biomass Precursors In Default Medium |

3 |

Blocked Biomass Precursors In Complete Medium |

3 |

Ratio of Direct Metabolites in Biomass Reaction |

0.12 |

Number of Missing Essential Biomass Precursors |

3 |

Energy Metabolism

Non-Growth Associated Maintenance Reaction |

1 |

Growth-associated Maintenance in Biomass Reaction |

true |

Number of Reversible Oxygen-Containing Reactions |

6 |

Erroneous Energy-generating Cycles | Info |

 MNXM3 |

Skipped |

 MNXM63 |

Skipped |

 MNXM51 |

Skipped |

 MNXM121 |

Skipped |

 MNXM423 |

Skipped |

 MNXM6 |

Skipped |

 MNXM10 |

Skipped |

 MNXM38 |

Skipped |

 MNXM208 |

Skipped |

 MNXM191 |

Skipped |

 MNXM223 |

Skipped |

 MNXM7517 |

Skipped |

 MNXM12233 |

Skipped |

 MNXM558 |

Skipped |

 MNXM21 |

Skipped |

 MNXM89557 |

Skipped |

Network Topology

Universally Blocked Reactions |

785 |

Orphan Metabolites |

212 |

Dead-end Metabolites |

195 |

Stoichiometrically Balanced Cycles |

360 |

Metabolite Production In Complete Medium |

878 |

Metabolite Consumption In Complete Medium |

924 |

Matrix Conditioning

Ratio Min/Max Non-Zero Coefficients |

0.00 |

Independent Conservation Relations |

308 |

Rank |

1365 |

Degrees Of Freedom |

431 |

Experimental Data Comparison

Growth Prediction |

Skipped |

Gene Essentiality Prediction |

Skipped |

Misc. Tests

Environment Python Version 3.6.12 Platform Linux Memote Version 0.11.1

Package Versions

{"click-log":"0.3.2","requests":"2.24.0","click":"7.1.2","travis-encrypt":"1.1.2","click-configfile":"0.2.3","importlib-resources":"3.0.0","pytest":"6.0.1","ruamel.yaml":"0.16.10","cobra":"0.18.1","goodtables":"2.5.0","equilibrator-api":"0.1.26","Jinja2":"2.11.2","six":"1.15.0","cookiecutter":"1.7.2","sqlalchemy":"1.3.19","future":"0.18.2","lxml":"4.5.2","gitpython":"3.1.7","depinfo":"1.5.4","numpydoc":"1.1.0","pylru":"1.2.0","pandas":"1.1.0","sympy":"1.6.2","memote":"0.11.1","pip":"20.2.2","setuptools":"49.6.0","wheel":"0.35.1"}
