# Supplementary material for: Modeling Drosophila gut microbe interactions reveals metabolic interconnectivity
Source: iScience. 2021 Oct 6;24(11):103216. doi: 10.1016/j.isci.2021.103216 (PMC8528732; doi:10.1016/j.isci.2021.103216)

Independent Section

Contains tests that are independent of the class of modeled organism, a model's

Consistency

Stoichiometric Consistency	99.9%	X3
Mass Balance	99.0%	
Charge Balance	99.9%	
Metabolite Connectivity	100.0%	
Unbounded Flux In Default Medium	68.0%	
Sub Total	95%	X3

Annotation - Metabolites

Presence of Metabolite Annotation	100.0%
Metabolite Annotations Per Database	Info
pubchem.compound	0.0%
kegg.compound	86.1%
seed.compound	100.0%
inchikey	81.2%
inchi	0.0%
chebi	87.0%
hmdb	59.4%
reactome	34.0%
metanetx.chemical	99.9%
bigg.metabolite	66.6%
biocyc	77.3%
Metabolite Annotation Conformity Per Database	Info
pubchem.compound	0.0%
kegg.compound	100.0%
seed.compound	100.0%
inchikey	100.0%
inchi	0.0%
chebi	100.0%

Specific Section

Covers general statistics and specific aspects of a metabolic network that are not

SBML

SBML Level and Version	Errored
FBC enabled	Errored

Basic Information

Model Identifier	Acetobacter_indonesiensis_A4
Total Metabolites	1,763
Total Reactions	1,931
Total Genes	631
Total Compartments	3
Metabolic Coverage	3.06

Metabolite Information

Unique Metabolites	1,618
Duplicate Metabolites in Identical Compartments	18
Metabolites without Charge	0
Metabolites without Formula	0
Medium Components	50

Reaction Information

Purely Metabolic Reactions	1,588
Purely Metabolic Reactions with Constraints	120
Transport Reactions	193
Transport Reactions with Constraints	3
Thermodynamic Reversibility of Purely Metabolic Reactions	0.40
Reactions With Partially Identical Annotations	0.01

metanetx.chemical	99.9%
bigg.metabolite	100.0%
biocyc	100.0%
Uniform Metabolite Identifier Namespace	100.0%
Sub Total	86%

Annotation - Reactions

Presence of Reaction Annotation	100.0%
Reaction Annotations Per Database	Info
rhea	0.0%
kegg.reaction	53.5%
seed.reaction	92.0%
metanetx.reaction	62.1%
bigg.reaction	39.4%
reactome	0.0%
ec-code	80.8%
brenda	0.0%
biocyc	42.1%
Reaction Annotation Conformity Per Database	Info
rhea	0.0%
kegg.reaction	100.0%
seed.reaction	100.0%
metanetx.reaction	100.0%
bigg.reaction	100.0%
reactome	0.0%
ec-code	99.6%
brenda	0.0%
biocyc	100.0%
Uniform Reaction Identifier Namespace	100.0%
Sub Total	77%

Annotation - Genes

Presence of Gene Annotation	0.0%

Gene-Protein-Reaction (GPR) Associations

Reactions without GPR	329
Fraction of Transport Reactions without GPR	0.40
Enzyme Complexes	78

Biomass

Biomass Reactions Identified	1
Biomass Consistency	Errored
Biomass Production In Default Medium	0.56
Unrealistic Growth Rate In Default Medium	false
Biomass Production In Complete Medium	109.42
Blocked Biomass Precursors In Default Medium	3
Blocked Biomass Precursors In Complete Medium	3
Ratio of Direct Metabolites in Biomass Reaction	0.12
Number of Missing Essential Biomass Precursors	3

Energy Metabolism

Non-Growth Associated Maintenance Reaction	1
Growth-associated Maintenance in Biomass Reaction	true
Number of Reversible Oxygen-Containing Reactions	6
Erroneous Energy-generating Cycles	Info
MNXM3	Skipped
MNXM63	Skipped
MNXM51	Skipped
MNXM121	Skipped

uniprot	0.0%	
ecogene	0.0%	
kegg.genes	0.0%	
ncbigi	0.0%	
ncbigene	0.0%	
ncbiprotein	0.0%	
ccds	0.0%	
hprd	0.0%	
asap	0.0%	
Gene Annotation Conformity Per Database	Info	
refseq	0.0%	
uniprot	0.0%	
ecogene	0.0%	
kegg.genes	0.0%	
ncbigi	0.0%	
ncbigene	0.0%	
ncbiprotein	0.0%	
ccds	0.0%	
hprd	0.0%	
asap	0.0%	
Sub Total	0%	

Annotation - SBO Terms

Metabolite General SBO Presence	100.0%
Metabolite SBO:0000247 Presence	100.0%
Reaction General SBO Presence	100.0%
Metabolic Reaction SBO:0000176 Presence	99.9%
Transport Reaction SBO:0000185 Presence	63.7%
Exchange Reaction SBO:0000627 Presence	100.0%
Demand Reaction SBO:0000628 Presence	100.0%
Sink Reactions SBO:0000632 Presence	Skipped
Gene General SBO Presence	0.0%

MNXM10	Skipped
MNXM38	Skipped
MNXM208	Skipped
MNXM191	Skipped
MNXM223	Skipped
MNXM7517	Skipped
MNXM12233	Skipped
MNXM558	Skipped
MNXM21	Skipped
MNXM89557	Skipped

Network Topology

Universally Blocked Reactions	837
Orphan Metabolites	209
Dead-end Metabolites	207
Stoichiometrically Balanced Cycles	265
Metabolite Production In Complete Medium	922
Metabolite Consumption In Complete Medium	959

Matrix Conditioning

Ratio Min/Max Non-Zero Coefficients	0.00
Independent Conservation Relations	321
Rank	1442
Degrees Of Freedom	489

Experimental Data Comparison

Growth Prediction	Skipped
Gene Essentiality Prediction	Skipped

Misc. Tests

SBO:0000629 Presence	[illegible]
Sub Total	69%
Total Score	77%

Platform	Linux
Memote Version	0.11.1

Total Score

77%

Score per Category
Export

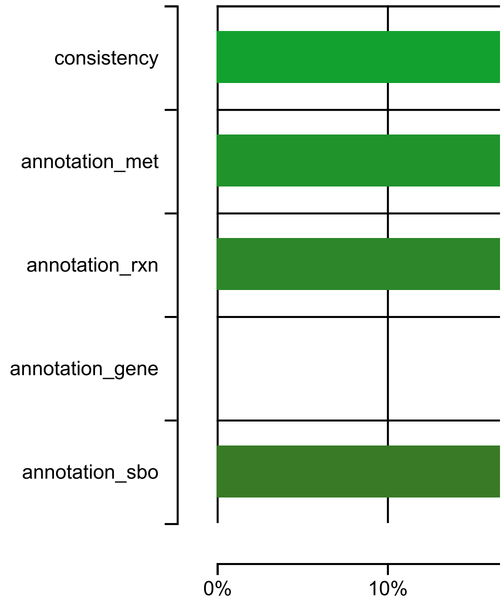

Supplement: Data S3. MEMOTE analysis results for the quality control measurements of the genome-scale model quality. The zip folder contains all individual results as PDF files as well as interactive versions, which can be accessed via the html files, related to Table 1 [file mmc4.zip › MEMOTE/Static-PDF-Version/MEMOTE-Acetobacter_indonesiensis_A4.pdf]
