# Supplementary material for: Modeling Drosophila gut microbe interactions reveals metabolic interconnectivity
Source: iScience. 2021 Oct 6;24(11):103216. doi: 10.1016/j.isci.2021.103216 (PMC8528732; doi:10.1016/j.isci.2021.103216)

Independent Section

Contains tests that are independent of the class of modeled organism, a model's [illegible]

Consistency

Stoichiometric Consistency	99.9%	X3
Mass Balance	99.0%	
Charge Balance	99.9%	
Metabolite Connectivity	100.0%	
Unbounded Flux In Default Medium	71.1%	
Sub Total	96%	X3

Annotation - Metabolites

Presence of Metabolite Annotation	100.0%
Metabolite Annotations Per Database	Info
pubchem.compound	0.0%
kegg.compound	86.0%
seed.compound	100.0%
inchikey	81.1%
inchi	0.0%
chebi	86.8%
hmdb	58.6%
reactome	33.1%
metanetx.chemical	99.9%
bigg.metabolite	66.5%
biocyc	77.9%
Metabolite Annotation Conformity Per Database	Info
pubchem.compound	0.0%
kegg.compound	100.0%
seed.compound	100.0%
inchikey	100.0%
inchi	0.0%
chebi	100.0%

Specific Section

Covers general statistics and specific aspects of a metabolic network that are not [illegible]

SBML

SBML Level and Version	Errored
FBC enabled	Errored

Basic Information

Model Identifier	Acetobacter_pasteurianus_B5
Total Metabolites	1,673
Total Reactions	1,796
Total Genes	580
Total Compartments	3
Metabolic Coverage	3.10

Metabolite Information

Unique Metabolites	1,536
Duplicate Metabolites in Identical Compartments	18
Metabolites without Charge	0
Metabolites without Formula	0
Medium Components	50

Reaction Information

Purely Metabolic Reactions	1,468
Purely Metabolic Reactions with Constraints	112
Transport Reactions	183
Transport Reactions with Constraints	3
Thermodynamic Reversibility of Purely Metabolic Reactions	0.40
Reactions With Partially Identical Annotations	0.01

metanetx.chemical	99.9%	
bigg.metabolite	100.0%	
biocyc	100.0%	
Uniform Metabolite Identifier Namespace	100.0%	
Sub Total	86%	

Annotation - Reactions

Presence of Reaction Annotation	100.0%	
Reaction Annotations Per Database	Info	
rhea	0.0%	
kegg.reaction	53.7%	
seed.reaction	91.9%	
metanetx.reaction	61.8%	
bigg.reaction	39.0%	
reactome	0.0%	
ec-code	80.3%	
brenda	0.0%	
biocyc	41.8%	
Reaction Annotation Conformity Per Database	Info	
rhea	0.0%	
kegg.reaction	100.0%	
seed.reaction	100.0%	
metanetx.reaction	100.0%	
bigg.reaction	100.0%	
reactome	0.0%	
ec-code	99.7%	
brenda	0.0%	
biocyc	100.0%	
Uniform Reaction Identifier Namespace	100.0%	
Sub Total	77%	

Annotation - Genes

Presence of Gene Annotation	0.0%	

Gene-Protein-Reaction (GPR) Associations

Reactions without GPR	319	
Fraction of Transport Reactions without GPR	0.43	
Enzyme Complexes	74	

Biomass

Biomass Reactions Identified	1	
Biomass Consistency	Errored	
Biomass Production In Default Medium	0.41	
Unrealistic Growth Rate In Default Medium	false	
Biomass Production In Complete Medium	65.13	
Blocked Biomass Precursors In Default Medium	3	
Blocked Biomass Precursors In Complete Medium	3	
Ratio of Direct Metabolites in Biomass Reaction	0.12	
Number of Missing Essential Biomass Precursors	3	

Energy Metabolism

Non-Growth Associated Maintenance Reaction	1	
Growth-associated Maintenance in Biomass Reaction	true	
Number of Reversible Oxygen-Containing Reactions	6	
Erroneous Energy-generating Cycles	Info	
MNXM3	Skipped	
MNXM63	Skipped	
MNXM51	Skipped	
MNXM121	Skipped	

[illegible]	
uniprot	0.0%
ecogene	0.0%
kegg.genes	0.0%
ncbigi	0.0%
ncbigene	0.0%
ncbiprotein	0.0%
ccds	0.0%
hprd	0.0%
asap	0.0%
Gene Annotation Conformity Per Database	Info
refseq	0.0%
uniprot	0.0%
ecogene	0.0%
kegg.genes	0.0%
ncbigi	0.0%
ncbigene	0.0%
ncbiprotein	0.0%
ccds	0.0%
hprd	0.0%
asap	0.0%
Sub Total	0%

Annotation - SBO Terms

Metabolite General SBO Presence	100.0%
Metabolite SBO:0000247 Presence	100.0%
Reaction General SBO Presence	100.0%
Metabolic Reaction SBO:0000176 Presence	99.9%
Transport Reaction SBO:0000185 Presence	62.8%
Exchange Reaction SBO:0000627 Presence	100.0%
Demand Reaction SBO:0000628 Presence	100.0%
Sink Reactions SBO:0000632 Presence	Skipped
Gene General SBO Presence	0.0%

MNXM10	Skipped
MNXM38	Skipped
MNXM208	Skipped
MNXM191	Skipped
MNXM223	Skipped
MNXM7517	Skipped
MNXM12233	Skipped
MNXM558	Skipped
MNXM21	Skipped
MNXM89557	Skipped

Network Topology

Universally Blocked Reactions	785
Orphan Metabolites	212
Dead-end Metabolites	195
Stoichiometrically Balanced Cycles	360
Metabolite Production In Complete Medium	878
Metabolite Consumption In Complete Medium	924

Matrix Conditioning

Ratio Min/Max Non-Zero Coefficients	0.00
Independent Conservation Relations	308
Rank	1365
Degrees Of Freedom	431

Experimental Data Comparison

Growth Prediction	Skipped
Gene Essentiality Prediction	Skipped

Misc. Tests

SBO:0000629 Presence	[illegible]
Sub Total	69% X2
Total Score	78%

Platform	Linux
Memote Version	0.11.1

Total Score

78%

Score per Category

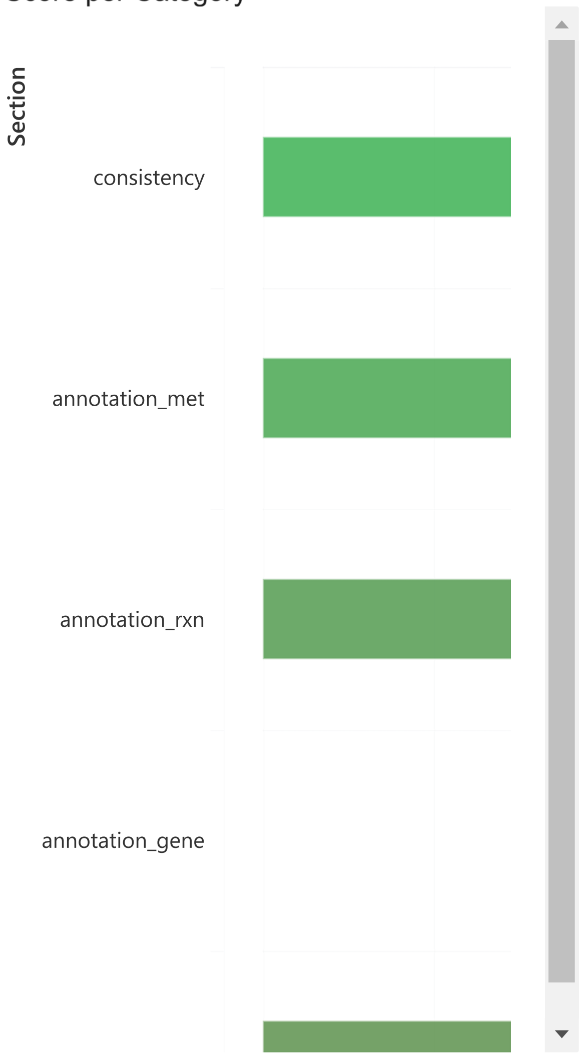

Supplement: Data S3. MEMOTE analysis results for the quality control measurements of the genome-scale model quality. The zip folder contains all individual results as PDF files as well as interactive versions, which can be accessed via the html files, related to Table 1 [file mmc4.zip › MEMOTE/Static-PDF-Version/MEMOTE-Acetobacter_pasteurianus_B5.pdf]
