# Supplementary material for: Modeling Drosophila gut microbe interactions reveals metabolic interconnectivity
Source: iScience. 2021 Oct 6;24(11):103216. doi: 10.1016/j.isci.2021.103216 (PMC8528732; doi:10.1016/j.isci.2021.103216)

Independent Section

Contains tests that are independent of the class of modeled organism, a model's

Specific Section

Covers general statistics and specific aspects of a metabolic network that are not

Consistency

Stoichiometric Consistency	100.0%	X3
Mass Balance	99.6%	
Charge Balance	99.9%	
Metabolite Connectivity	100.0%	
Unbounded Flux In Default Medium	78.7%	
Sub Total	97%	X3

Annotation - Metabolites

Presence of Metabolite Annotation	100.0%
Metabolite Annotations Per Database	Info
pubchem.compound	0.0%
kegg.compound	81.9%
seed.compound	99.7%
inchikey	75.7%
inchi	0.0%
chebi	83.1%
hmdb	57.8%
reactome	36.0%
metanetx.chemical	99.7%
bigg.metabolite	66.8%
biocyc	75.8%
Metabolite Annotation Conformity Per Database	Info
pubchem.compound	0.0%
kegg.compound	100.0%
seed.compound	100.0%
inchikey	100.0%
inchi	0.0%
chebi	100.0%

SBML

SBML Level and Version	Errored
FBC enabled	Errored

Basic Information

Model Identifier	Lactobacillus_brevis_B6
Total Metabolites	1,411
Total Reactions	1,584
Total Genes	473
Total Compartments	3
Metabolic Coverage	3.35

Metabolite Information

Unique Metabolites	1,257
Duplicate Metabolites in Identical Compartments	17
Metabolites without Charge	0
Metabolites without Formula	0
Medium Components	50

Reaction Information

Purely Metabolic Reactions	1,203
Purely Metabolic Reactions with Constraints	70
Transport Reactions	218
Transport Reactions with Constraints	2
Thermodynamic Reversibility of Purely Metabolic Reactions	0.28
Reactions With Partially Identical Annotations	0.01

metanetx.chemical	99.9%	
bigg.metabolite	100.0%	
biocyc	100.0%	
Uniform Metabolite Identifier Namespace	100.0%	
Sub Total	86%	

Annotation - Reactions

Presence of Reaction Annotation	100.0%	
Reaction Annotations Per Database	Info	
rhea	0.0%	
kegg.reaction	44.6%	
seed.reaction	89.1%	
metanetx.reaction	53.5%	
bigg.reaction	35.3%	
reactome	0.0%	
ec-code	71.8%	
brenda	0.0%	
biocyc	36.5%	
Reaction Annotation Conformity Per Database	Info	
rhea	0.0%	
kegg.reaction	100.0%	
seed.reaction	100.0%	
metanetx.reaction	100.0%	
bigg.reaction	100.0%	
reactome	0.0%	
ec-code	99.4%	
brenda	0.0%	
biocyc	100.0%	
Uniform Reaction Identifier Namespace	100.0%	
Sub Total	76%	

Annotation - Genes

Presence of Gene Annotation	0.0%	

Gene-Protein-Reaction (GPR) Associations

Reactions without GPR	263	
Fraction of Transport Reactions without GPR	0.26	
Enzyme Complexes	60	

Biomass

Biomass Reactions Identified	1	
Biomass Consistency	Errored	
Biomass Production In Default Medium	0.07	
Unrealistic Growth Rate In Default Medium	false	
Biomass Production In Complete Medium	110.74	
Blocked Biomass Precursors In Default Medium	6	
Blocked Biomass Precursors In Complete Medium	6	
Ratio of Direct Metabolites in Biomass Reaction	0.15	
Number of Missing Essential Biomass Precursors	1	

Energy Metabolism

Non-Growth Associated Maintenance Reaction	1	
Growth-associated Maintenance in Biomass Reaction	true	
Number of Reversible Oxygen-Containing Reactions	2	
Erroneous Energy-generating Cycles	Info	
MNXM3	Skipped	
MNXM63	Skipped	
MNXM51	Skipped	
MNXM121	Skipped	
MNXM423	Skipped	

uniprot	0.0%
ecogene	0.0%
kegg.genes	0.0%
ncbigi	0.0%
ncbigene	0.0%
ncbiprotein	0.0%
ccds	0.0%
hprd	0.0%
asap	0.0%
Gene Annotation Conformity Per Database	Info
refseq	0.0%
uniprot	0.0%
ecogene	0.0%
kegg.genes	0.0%
ncbigi	0.0%
ncbigene	0.0%
ncbiprotein	0.0%
ccds	0.0%
hprd	0.0%
asap	0.0%
Sub Total	0%

Annotation - SBO Terms

Metabolite General SBO Presence	100.0%
Metabolite SBO:0000247 Presence	100.0%
Reaction General SBO Presence	100.0%
Metabolic Reaction SBO:0000176 Presence	99.8%
Transport Reaction SBO:0000185 Presence	64.2%
Exchange Reaction SBO:0000627 Presence	100.0%
Demand Reaction SBO:0000628 Presence	100.0%
Sink Reactions SBO:0000632 Presence	Skipped
Gene General SBO Presence	0.0%

MNXM38	Skipped
MNXM208	Skipped
MNXM191	Skipped
MNXM223	Skipped
MNXM7517	Skipped
MNXM12233	Skipped
MNXM558	Skipped
MNXM21	Skipped
MNXM89557	Skipped

Network Topology

Universally Blocked Reactions	649
Orphan Metabolites	178
Dead-end Metabolites	147
Stoichiometrically Balanced Cycles	245
Metabolite Production In Complete Medium	721
Metabolite Consumption In Complete Medium	709

Matrix Conditioning

Ratio Min/Max Non-Zero Coefficients	0.00
Independent Conservation Relations	257
Rank	1154
Degrees Of Freedom	430

Experimental Data Comparison

Growth Prediction	Skipped
Gene Essentiality Prediction	Skipped

Misc. Tests

Environment

SBO:0000629 Presence

Sub Total	69%
Total Score	78%

Total Score

78%

Score per Category

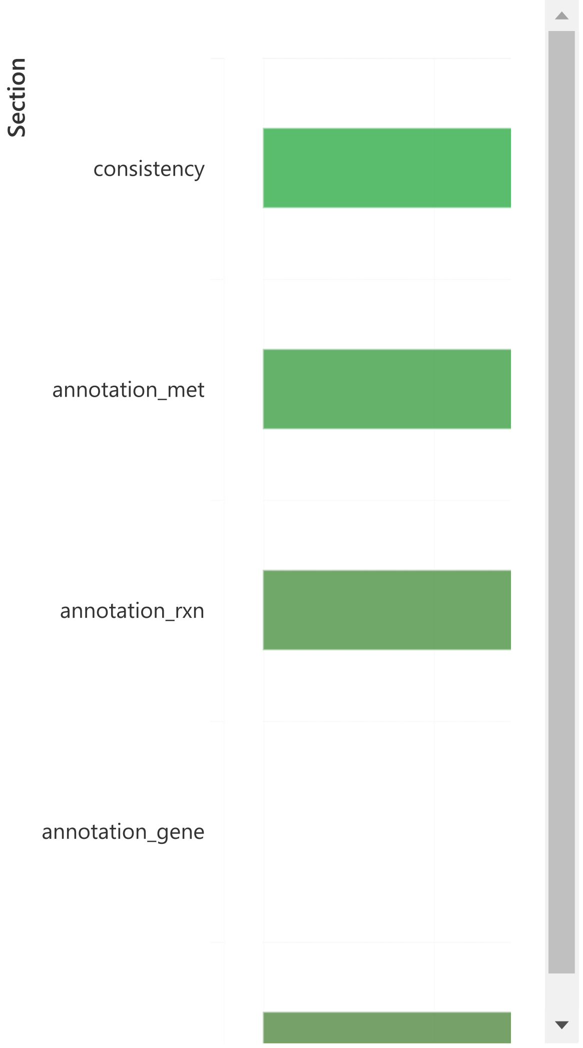

Supplement: Data S3. MEMOTE analysis results for the quality control measurements of the genome-scale model quality. The zip folder contains all individual results as PDF files as well as interactive versions, which can be accessed via the html files, related to Table 1 [file mmc4.zip › MEMOTE/Static-PDF-Version/MEMOTE-Lactobacillus_brevis_B6.pdf]
