# Supplementary material for: Modeling Drosophila gut microbe interactions reveals metabolic interconnectivity
Source: iScience. 2021 Oct 6;24(11):103216. doi: 10.1016/j.isci.2021.103216 (PMC8528732; doi:10.1016/j.isci.2021.103216)

# Independent Section

Contains tests that are independent of the class of modeled organism, a model's

## Consistency

| | | |
|---|---|---|
| Stoichiometric Consistency | 100.0% | X3 |
| Mass Balance | 99.5% | |
| Charge Balance | 100.0% | |
| Metabolite Connectivity | 100.0% | |
| Unbounded Flux In Default Medium | 79.0% | |
| Sub Total | 97% | X3 |

## Annotation - Metabolites

| | |
|---|---|
| Presence of Metabolite Annotation | 100.0% |
| Metabolite Annotations Per Database | Info |
| pubchem.compound | 0.0% |
| kegg.compound | 83.0% |
| seed.compound | 99.7% |
| inchikey | 77.3% |
| inchi | 0.0% |
| chebi | 84.2% |
| hmdb | 59.1% |
| reactome | 34.5% |
| metanetx.chemical | 99.7% |
| bigg.metabolite | 68.3% |
| biocyc | 76.1% |
| Metabolite Annotation Conformity Per Database | Info |
| pubchem.compound | 0.0% |
| kegg.compound | 100.0% |
| seed.compound | 100.0% |
| inchikey | 100.0% |
| inchi | 0.0% |
| chebi | 100.0% |

# Specific Section

Covers general statistics and specific aspects of a metabolic network that are not

## SBML

| | |
|---|---|
| SBML Level and Version | Errored |
| FBC enabled | Errored |

## Basic Information

| | |
|---|---|
| Model Identifier | Lactobacillus_plantarum_B2 |
| Total Metabolites | 1,567 |
| Total Reactions | 1,815 |
| Total Genes | 658 |
| Total Compartments | 3 |
| Metabolic Coverage | 2.76 |

## Metabolite Information

| | |
|---|---|
| Unique Metabolites | 1,411 |
| Duplicate Metabolites in Identical Compartments | 17 |
| Metabolites without Charge | 0 |
| Metabolites without Formula | 0 |
| Medium Components | 50 |

## Reaction Information

| | |
|---|---|
| Purely Metabolic Reactions | 1,414 |
| Purely Metabolic Reactions with Constraints | 91 |
| Transport Reactions | 232 |
| Transport Reactions with Constraints | 3 |
| Thermodynamic Reversibility of Purely Metabolic Reactions | 0.33 |
| Reactions With Partially Identical Annotations | 0.01 |

| | |
|---|---|
| metanetx.chemical | 99.9% |
| bigg.metabolite | 100.0% |
| biocyc | 100.0% |
| Uniform Metabolite Identifier Namespace | 100.0% |
| Sub Total | 86% |

## Annotation - Reactions

| | |
|---|---|
| Presence of Reaction Annotation | 100.0% |
| Reaction Annotations Per Database | Info |
| rhea | 0.0% |
| kegg.reaction | 47.6% |
| seed.reaction | 89.9% |
| metanetx.reaction | 56.5% |
| bigg.reaction | 37.2% |
| reactome | 0.0% |
| ec-code | 75.8% |
| brenda | 0.0% |
| biocyc | 37.9% |
| Reaction Annotation Conformity Per Database | Info |
| rhea | 0.0% |
| kegg.reaction | 100.0% |
| seed.reaction | 100.0% |
| metanetx.reaction | 100.0% |
| bigg.reaction | 100.0% |
| reactome | 0.0% |
| ec-code | 99.6% |
| brenda | 0.0% |
| biocyc | 100.0% |
| Uniform Reaction Identifier Namespace | 100.0% |
| Sub Total | 76% |

## Annotation - Genes

| | |
|---|---|
| Presence of Gene Annotation | 0.0% |

# Gene-Protein-Reaction (GPR) Associations

| | |
|---|---|
| Reactions without GPR | 284 |
| Fraction of Transport Reactions without GPR | 0.22 |
| Enzyme Complexes | 80 |

# Biomass

| | |
|---|---|
| Biomass Reactions Identified | 1 |
| Biomass Consistency | Errored |
| Biomass Production In Default Medium | 0.42 |
| Unrealistic Growth Rate In Default Medium | false |
| Biomass Production In Complete Medium | 105.22 |
| Blocked Biomass Precursors In Default Medium | 6 |
| Blocked Biomass Precursors In Complete Medium | 6 |
| Ratio of Direct Metabolites in Biomass Reaction | 0.13 |
| Number of Missing Essential Biomass Precursors | 1 |

# Energy Metabolism

| | |
|---|---|
| Non-Growth Associated Maintenance Reaction | 1 |
| Growth-associated Maintenance in Biomass Reaction | true |
| Number of Reversible Oxygen-Containing Reactions | 2 |
| Erroneous Energy-generating Cycles | Info |
| MNXM3 | Skipped |
| MNXM63 | Skipped |
| MNXM51 | Skipped |
| MNXM121 | Skipped |
| MNXM423 | Skipped |

| | |
|---|---|
| [illegible] | |
| uniprot | 0.0% |
| ecogene | 0.0% |
| kegg.genes | 0.0% |
| ncbigi | 0.0% |
| ncbigene | 0.0% |
| ncbiprotein | 0.0% |
| ccds | 0.0% |
| hprd | 0.0% |
| asap | 0.0% |
| Gene Annotation Conformity Per Database | Info |
| refseq | 0.0% |
| uniprot | 0.0% |
| ecogene | 0.0% |
| kegg.genes | 0.0% |
| ncbigi | 0.0% |
| ncbigene | 0.0% |
| ncbiprotein | 0.0% |
| ccds | 0.0% |
| hprd | 0.0% |
| asap | 0.0% |
| Sub Total | 0% |

## Annotation - SBO Terms

| | |
|---|---|
| Metabolite General SBO Presence | 100.0% |
| Metabolite SBO:0000247 Presence | 100.0% |
| Reaction General SBO Presence | 100.0% |
| Metabolic Reaction SBO:0000176 Presence | 99.9% |
| Transport Reaction SBO:0000185 Presence | 60.8% |
| Exchange Reaction SBO:0000627 Presence | 100.0% |
| Demand Reaction SBO:0000628 Presence | 100.0% |
| Sink Reactions SBO:0000632 Presence | Skipped |
| Gene General SBO Presence | 0.0% |

| | |
|---|---|
| MNXM38 | Skipped |
| MNXM208 | Skipped |
| MNXM191 | Skipped |
| MNXM223 | Skipped |
| MNXM7517 | Skipped |
| MNXM12233 | Skipped |
| MNXM558 | Skipped |
| MNXM21 | Skipped |
| MNXM89557 | Skipped |

## Network Topology

| | |
|---|---|
| Universally Blocked Reactions | 727 |
| Orphan Metabolites | 201 |
| Dead-end Metabolites | 165 |
| Stoichiometrically Balanced Cycles | 278 |
| Metabolite Production In Complete Medium | 783 |
| Metabolite Consumption In Complete Medium | 785 |

## Matrix Conditioning

| | |
|---|---|
| Ratio Min/Max Non-Zero Coefficients | 0.00 |
| Independent Conservation Relations | 262 |
| Rank | 1305 |
| Degrees Of Freedom | 510 |

## Experimental Data Comparison

| | |
|---|---|
| Growth Prediction | Skipped |
| Gene Essentiality Prediction | Skipped |

## Misc. Tests

## Environment

SBO:0000629 Presence

Sub Total 69%

Total Score 78%

Total Score

# 78%

Score per Category

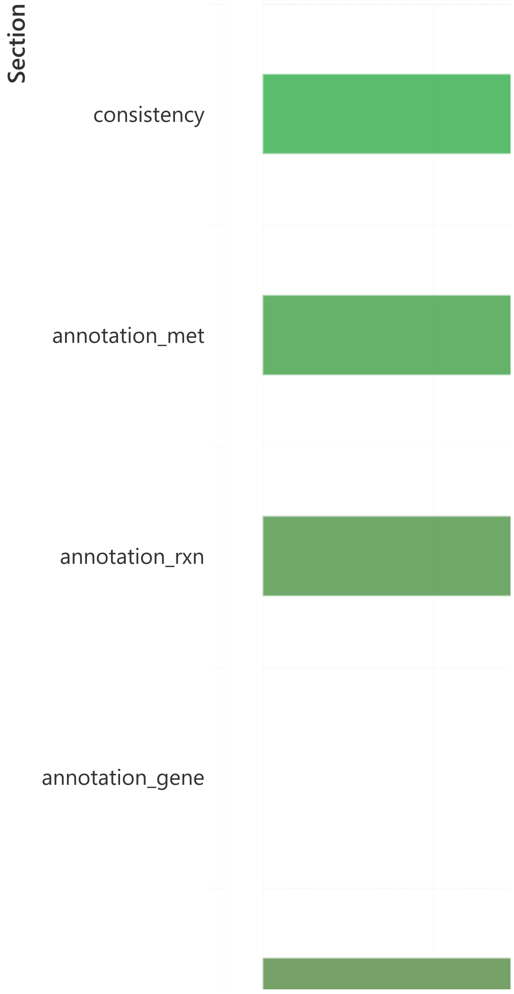

Supplement: Data S3. MEMOTE analysis results for the quality control measurements of the genome-scale model quality. The zip folder contains all individual results as PDF files as well as interactive versions, which can be accessed via the html files, related to Table 1 [file mmc4.zip › MEMOTE/Static-PDF-Version/MEMOTE-Lactobacillus_plantarum_B2.pdf]
